# Asymmetric Synthesis of Photophore-Containing Lactisole Derivatives to Elucidate Sweet Taste Receptors

**DOI:** 10.3390/molecules25122790

**Published:** 2020-06-17

**Authors:** Tomoya Nakagita, Akiko Ishida, Zetryana Puteri Tachrim, Lei Wang, Takumi Misaka, Makoto Hashimoto

**Affiliations:** 1 Department of Applied Biological Chemistry, Graduate School of Agricultural and Life Sciences, The University of Tokyo, Tokyo 113-8657, Japan; nakagita.tomoya.eo@ehime-u.ac.jp (T.N.); amisaka@mail.ecc.u-tokyo.ac.jp (T.M.); 2 Proteo-Science Center, Ehime University, Ehime 791-8577, Japan; 3 Division of Applied Bioscience, Graduate School of Agriculture, Hokkaido University, Kita 9, Nishi 9, Kita-ku, Sapporo 060-8589, Japan; a-ishida@frontier.hokudai.ac.jp (A.I.); zetry@chem.itb.ac.id (Z.P.T.); leiwang@dlut.edu.cn (L.W.); 4 Program Study of Chemistry, Faculty of Mathematics and Natural Sciences, Institut Teknologi Bandung, Jalan Ganesha 10, Bandung 40132, Indonesia; 5 Department of Pharmacy, School of Chemical Engineering, Dalian University of Technology, Dalian 116023, China

**Keywords:** lactisole, photoaffinity label, sweet taste, diazirine, azide, benzophenone

## Abstract

Lactisole, which has a 2-phenoxy propionic acid skeleton, is well-known as an inhibitor of sweet taste receptors. We recently revealed some of the structure–activity relationships of the aromatic ring and chiral center of lactisole. Photoaffinity labeling is one of the common chemical biology methods to elucidate the interaction between bioactive compounds and biomolecules. In this paper, the novel asymmetric synthesis of lactisole derivatives with common photophores (benzophenone, azide and trifluoromethyldiazirine) for photoaffinity labeling is described. The synthetic compounds are subjected to cell-based sweet taste receptors, and the substitution with trifluoromethyldiazirinyl photophore shows the highest affinity to the receptor of the synthesized compounds.

## 1. Introduction

Lactisole (2-(4-methoxyphenoxy)propanoic acid) [1,2] is well-known as an inhibitor of sweet taste receptors and has been shown to interact with the transmembrane domain of the T1R3 subunit (T1R3-TMD) of the receptor [3]. The mother skeleton, 2-phenoxypropanoic acid, is found in several phenoxy herbicides, such as 2-(2,4-dichlorophenoxy)propanoic acid (dichlorprop, 2,4-DP) 2-(4-chlorophenoxy)propanoic acid (4-CPP) [4], and (*R*)-isomers of them have herbicide activities. We recently reported that the (*S*)-isomers at the 2-position of lactisole and 2,4-DP at the 2-position have higher affinity for sweet taste receptor than (*R*)-isomers [5]. These results are inconsistent with the herbicide activity for 2-phenoxypropanoic acid [6]. The detailed functional analysis of the stereocenter may be important to elucidate sweet taste receptors.

**Figure 1:**
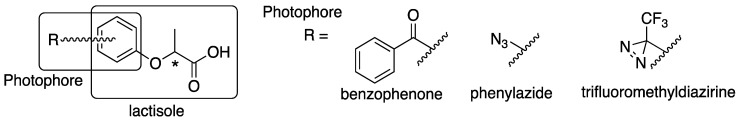
Structures of photoreactive lactisole derivatives in this study.

Photoaffinity labeling [7,8,9,10] is a useful biochemical method for the analysis of biological interactions between low-molecular-weight bioactive compounds and biomolecules. The methodology may afford other information near the binding sites which cannot be obtained from structure–activity relationship studies. Three major photophores—phenylazide, benzophenone and (trifluoromethyl)phenyldiazirine—are used for photoaffinity labeling [11]. We reported several preparations of gustatory ligands to elucidate gustatory receptors [12,13,14,15,16,17,18,19,20]; however, there are few studies on the synthesis of photoreactive lactisole derivatives which have aimed to elucidate their biological activities. In this paper, we describe the comprehensive synthesis of photoreactive lactisole derivatives (Figure 1), which we then used in an inhibitory activity assay for cell-based human sweet taste receptors.

## 2. Results

### 2.1. Synthesis

The key steps to construct a lactisole skeleton in an asymmetric manner are achieved by the Mitsunobu reaction for optically pure methyl lactate and phenol derivatives of photophore precursors. Details for each photophore are described below.

#### 2.1.1. Benzophenone-Based Lactisole Derivatives

**Figure 2:**
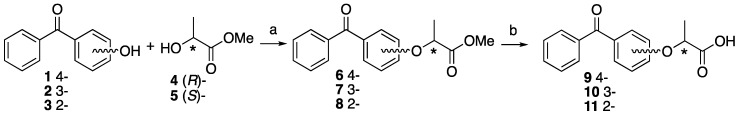
Synthesis of benzophenone-containing lactisole derivatives. (**a**) (1) PPh_3_, CH_2_Cl_2_, 0 °C, 10 min; (2) diethyl azodicarboxylate (DEAD), CH_2_Cl_2_, rt, 8 h, (*S*)-**6** 88%, (*R*)-**6** quant., (*S*)-**7** quant., (*R*)-**7** quant, (*S*)-**8** 88%, (*R*)-**8** 82%; (**b**) K_2_CO_3_, CH_3_OH, H_2_O, reflux, 2 h, (*S*)-**9** quant, (*R*)-**9** quant., (*S*)-**10** 80%, (*R*)-**10** quant, (*S*)-**11** 87%, (*R*)-**11** 88%.

A few reports have described the preparations of racemic benzophenone-based lactisole derivatives and their separation by chromatography [21,22,23,24,25]. However, to date, the asymmetric synthesis of benzophenone-modified lactisole derivatives has not been reported. Hydroxybenzophenone derivatives (**1**–**3**), optically pure methyl lactate (**4** and **5**, 1.5 equiv.) and triphenylphosphine (1.2 equiv.) were preincubated at 0 °C for 10 min in dichloromethane and diethyl azodicarboxylate (1.5 equiv.) was slowly added to the solution. The reaction mixture was stirred at room temperature overnight and worked up with a general procedure. Purification with column chromatography afforded the optically pure methyl 2-benzoylphenoxypropionate derivatives **6**–**8** with a good yield. The methyl esters were subjected to chiral high-performance liquid chromatography (HPLC) with Chiralpak IG [2] to ensure the configuration of the chiral center. The methyl esters were hydrolyzed under reflux in the presence of K_2_CO_3_ to afford derivatives **9**–**13** in high yields (Figure 2).

#### 2.1.2. Azide-Based Lactisole Derivatives

**Figure 3:**
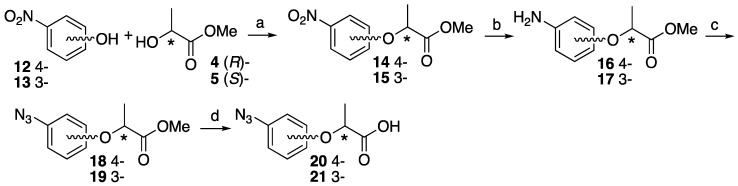
Synthesis of 4- and 3- azide-substituted lactisole derivatives. (**a**) (1) PPh_3_, CH_2_Cl_2_, 0 °C, 10 min; (2) DEAD, CH_2_Cl_2_, rt, 8 h, (*S*)-**14** 99%, (*R*)-**14** quant., (*S*)-**15** quant., (*R*)-**15** quant, (**b**) H_2_, Pd/C, CH_3_OH, rt, 2 h, (*S*)-**16** quant., (*R*)-**16** quant., (*S*)-**17** quant., (*R*)-**17** 87%, (**c**) (1) NaNO_2_, HCl, H_2_O, 0 °C; (2) NaN_3_, 0 °C, 30 min, then rt, 30 min, (*S*)-**18** quant., (*R*)-**18** 93%., (*S*)-**19** 92%., (*R*)-**19** 85%, (**d**) NaOH, CH_3_OH, H_2_O, reflux, 5 h, (*S*)-**20** 91%, (*R*)-**20** 96%, (*S*)-**21** quant., (*R*)-**21** 81%.

An example of the synthesis of racemic azide-based lactisole ethyl ester has been reported previously [26], but to date, the asymmetric synthesis of azide modified on the aromatic ring of the lactisole skeleton has not been reported. Akazome et al. reported the asymmetric synthesis of (*R*)-2-nitro (*R*)-**23** [27] and (*R*)-3-nitro (*R*)-**15** [28,29]-substituted lactisole methyl ester. Our retrosynthesis for comprehensive preparations of azide-based lactisole derivatives was based on the asymmetric synthesis of nitro-substituted lactisole skeletons, followed by the conversion of the nitro group to azide derivatives. First, 4- and 3- Nitrophenol **12** and **13**, and optically pure methyl lactate **4** and **5** (1.5 equiv.) and triphenylphosphine (1.2 equiv.) were preincubated at 0 °C for 10 min in dichloromethane. Diethyl azodicarboxylate (1.5 equiv.) was slowly added to the solution to construct nitro-substituted methyl lactisole **14** and **15**. Nitro groups were reduced under a hydrogen atmosphere in the presence of 5% Pd/C to afford amino derivatives **16** and **17**. The aniline derivatives were subjected to diazotization followed by azidation to obtain azide-substituted methyl lactisole derivatives **18** and **19**, which were checked by chiral center configuration. The methyl esters were hydrolyzed with sodium hydroxide under reflux condition to afford **20** and **21** in high yields (Figure 3).

Then, 2-Nitrophenol **22** was also subjected to the Mitsunobu reaction in an identical manner with another isomer to make nitro methyl lactate skeleton **23**. The hydrogenation of the nitro group promoted subsequent intramolecular cyclization between the amino and methyl ester to afford 2-methyl-2*H*-benzo[*b*][1,4]oxazin-3(4*H*)-one as the sole product [27]. To prevent intramolecular cyclization, the methyl ester hydrolyzed **24**, then converted to *tert*-butyl ester **25**. The hydrogenation of nitro group of **25** proceeded smoothly to isolate aniline derivative **26** within an hour. Although *tert*-butyl ester was selected, intramolecular cyclization between the aniline and ester proceeded with a longer reaction time (>4 h). Compound **26** was subjected to diazotization followed by azidation to construct phenylazide moiety **27**, which was checked by chiral center configuration.

**Figure 4:**
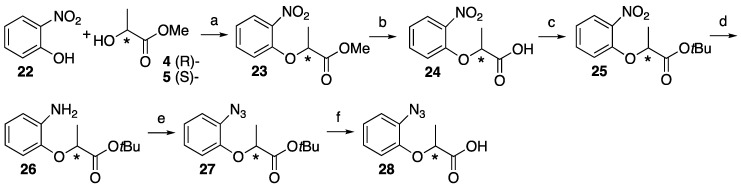
Synthesis of 2-azide-substituted lactisole derivatives. (**a**) (1) PPh_3_, CH_2_Cl_2_, 0 °C, 10 min; (2) DEAD, CH_2_Cl_2_, rt, 8 h, (*S*)-**23** quant., (*R*)-**23** quant., (**b**) NaOH, CH_3_OH, reflux, 1h, (*S*)-**24** quant., (*R*)-**24** quant., (**c**) *t*-Butyl bromide, K_2_CO_3_, tetrabutylammonium bromide, dimethyl acetamide, 55 °C, 2.5 h, (*S*)-**25** 77%, (*R*)-**25** 82%, (**d**) H_2_, Pd/C, CH_3_OH, rt, 1 h, (*S*)-**26** 73%, (*R*)-**26** 89%, (**e**) NaNO_2_, HCl, H_2_O, 0 °C; (2) NaN_3_, 0 °C, 30 min, then rt, 30 min, (*S*)-**27** quant., (*R*)-**27** 89%, (**f**) 4 M HCl, dioxane, rt, 1 h, (*S*)-**28** 68%, (*R*)-**26** 65%.

The deprotection of *tert*-butyl group of **27** with trifluoroacetic acid in dichloromethane afforded a complex mixture because the azide group was not stable under this condition. Mild acidic deprotection with 4 M HCl in dioxane was acceptable, but the partial decomposition of starting material **27** was observed over an hour. The starting material **27** could be recovered by partition within 1 h, and the hydrolysis was repeated for the recovered **27** three times to afford compound **28** in a moderate yield (Figure 4). The reaction with 2-nitrophenol **22** and optical pure *tert*-butyl lactate was also conducted to construct compound **25** directly, but unfortunately, the Mitsunobu reaction did not proceed for the *tert*-butyl lactate.

#### 2.1.3. Trifluotromethyldiazirine-Based Lactisole Derivatives

**Figure 5:**
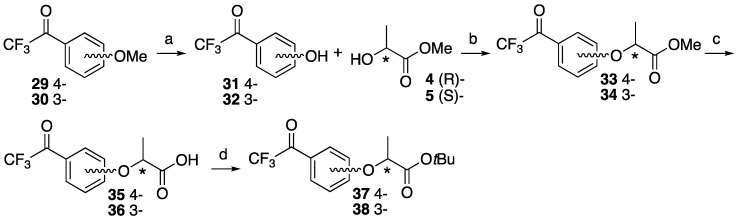
Synthesis of 3- and 4-trifluoroacetyl lactisole derivatives. (**a**) LiCl, DMF, reflux, 5 h for **31** 82%, BBr_3_, CH_2_Cl_2_, rt, 2 h for **32** quant. (**b**) (1) PPh_3_, CH_2_Cl_2_, 0 °C, 10 min; (2) DEAD, CH_2_Cl_2_, rt, 8 h, (*S*)-**33** 78%, (*R*)-**33** 74%, (*S*)-**34** 71%, (*R*)-**34** 75%. (**c**) K_2_CO_3_, MeOH, H_2_O, reflux, 2 h, (*S*)-**35** quant., (*R*)-**35** 95%, (*S*)-**36** 99%, (*R*)-**36** 97%. (**d**) *t*-BuBr, K_2_CO_3_, tetrabutylammonium bromide (TBAB), DMA, 55 °C, 5 h, (*S*)-**37** 66%, (*R*)-**37** 70%, (*S*)-**38** 38%, (*R*)-**38** 37%.

To date, trifluoromethyldiazirine-based lactisole derivatives have not been reported. The three-membered azi (N=N) partial structure of trifluoromethyldiazirine is not stable for the Mitsunobu condition because the reactant, diethyl azodicarboxylate DEAD also has an azo group in its structure. The reaction with diazirinyl phenol derivatives and 1′-hydroxy peracetylsucrose with DEAD or Tsuda reagents did not occur in our previous study [14]. The synthetic plan was based on the preparation of trifluoroacetyl modified on the aromatic ring of lactisole; then we constructed a diazirinyl three-membered ring on the trifluoroacetyl group. The reagent of liquid ammonia is essential to the construction of the diazirine moiety, but the reagent also reacts with methyl ester to form amide. *tert*-Butyl ester was utilized for this purpose. *p*-Trifluoroacetyl anisole and *m*-trifluoroacetyl anisole **29** and **30** were selected as the starting material for 4 and 3-substituted lactisoles. Compound **29** was treated with lithium chloride under a reflux condition to obtain trifluoroacetyl phenol **31** [30,31]. *m*-Trifluoroacetyl phenol **32** was synthesized with a boron tribromide treatment of **30** at room temperature [32]. Mitsunobu reactions for these phenols with chiral methyl lactate were archived in an identical manner to construct lactisole methyl ester skeletons **33** and **34**. After the hydrolysis of methyl esters with K_2_CO_3_ under the reflux condition, corresponding trifluoroacetyl-substituted carboxylic acids **35** and **36** were subjected to diazirine formation. However, it was difficult to construct diazirine moiety for **35** and **36** directly. The carboxylic acids were converted to *tert*-butyl esters with *tert*-butyl bromide in the presence of K_2_CO_3_ and tetrabutylammonium bromide (TBAB) under reflux conditions. Although the reactions were not completed within 5 h, the decompositions of the diazirinyl moiety of the starting material were observed over 5 h. *tert*-Butyl derivatives **37** and **38** were obtained with moderate yields and the starting materials could be recovered from the reaction mixture (Figure 5). The *tert*-butyl thioester formations [33] were also conducted for the carboxylic acids to improve the chemical yields of the protection. The chemical yields were almost identical with *tert*-butyl ester, but the recovery of the starting material was very difficult from the reaction mixture. Mitsunobu reactions for trifluoroacetyl phenol **31** and **32** with chiral *tert*-butyl lactate were also conducted, but no reactions were observed.

**Figure 6:**
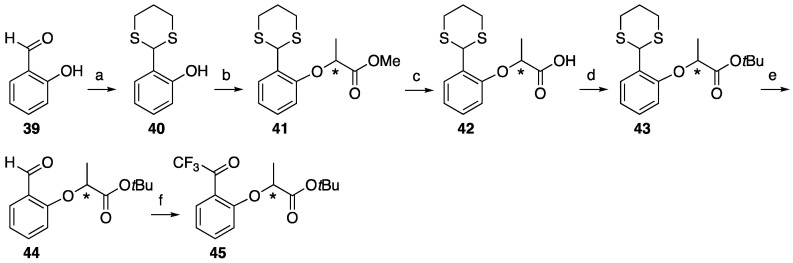
Synthesis of 2-trifluoroacetyl lactisole derivative. (**a**) 1,3-propanedithiol, I_2_, CH_2_Cl_2_, rt, 1 h, 95%, (**b**) (1) **4** or **5**, PPh_3_, CH_2_Cl_2_, 0 °C, 10 min; (2) DEAD, CH_2_Cl_2_, rt, 8 h, (*S*)-**41** 98%, (*R*)-**41** 98%, (**c**) K_2_CO_3_, MeOH, H_2_O, reflux, 1 h, (*S*)-**42** quant., (*R*)-**42** quant, (**d**) *t*-BuBr, K_2_CO_3_, TBAB, DMA, 55 °C, 2.5 h, (*S*)-**43** 83%, (*R*)-**43** 82%, (**e**) CH_3_I, NaHCO_3_, CH_3_CN, H_2_O, rt, 24 h, (*S*)-**44** 78%, (*R*)-**44** 79%, (**f**) (1) TMS-CF_3_, K_2_CO_3_, DMF, rt, 4 h, (2) 1 M HCl, rt, 4 h; (3) Dess–Martin periodinane, CH_2_Cl_2_, rt, 3 h, (*S*)-**45** 85%, (*R*)-**45** 89%.

Then, 2-trifluoroactyl phenol, which was prepared by the Fries rearrangement of phenyl 2,2,2-trifluoroacetate with AlCl_3_ [34], was subjected to Mitsunobu reaction with chiral methyl lactate **4** and did not afford a 2-trifluoroacetyl lactisole skeleton. The Mitsunobu reaction of salicylaldehyde and methyl lactate **4** followed by the conversion of aldehyde to the trifluoroacetyl group also failed due to the instability of methyl ester for the condition of trifluoroacetyl construction. Protected salicylaldehyde was selected for trifluoroacetyl substitution at the 2-position of lactisole. The carbonyl group was protected by thioacetal **40** [35], then subjected to a Mitsunobu reaction with chiral methyl lactate **4** and **5** to construct lactisole methyl ester **41** in an identical manner to that described above. After the hydrolysis of methyl esters with methanolic NaOH under the reflux condition, carboxylic acid **42** was converted to lactisole *tert*-butyl ester **43**, then thioacetal was deprotected with methyl iodide. Aldehyde **44** was treated with TMS-CF_3_ followed by oxidation with Dess–Martin periodinane [20] to afford 2-trifluoroacetyl-substituted lactisole *tert*-butyl ester **45** (Figure 6).

**Figure 7:**
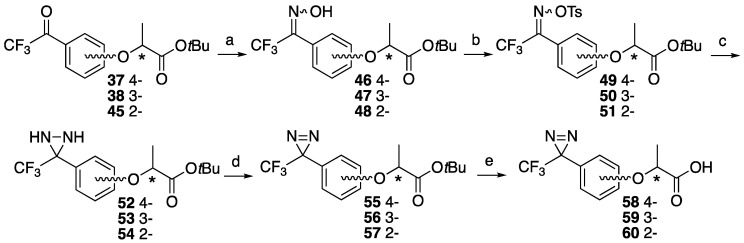
Synthesis of 2-, 3- and 4-trifluoromethyl diazirinyl lactisole derivatives. (**a**) NH_2_OH HCl, pyridine, EtOH, 60 °C, 12 h, (*S*)-**46** quant., (*R*)-**46** quant., (*S*)-**47** quant., (*R*)-**47** 85%, (*S*)-**48** quant., (*R*)-**48** 98%, (**b**) Tosyl chloride, DMAP, CH_2_Cl_2_, rt, 45 min, (*S*)-**49** quant., (*R*)-**49** 98%, (*S*)-**50** quant., (*R*)-**50** 93%, (*S*)-**51** 94%, (*R*)-**51** quant., (**c**) NH_3_ (l), Et_2_O, rt, 12 h, (*S*)-**52** 88%, (*R*)-**52** 88%, (*S*)-**53** 92%, (*R*)-**53** quant., (*S*)-**54** 76%, (*R*)-**54** 80%, (d) MnO_2_, CH_2_Cl_2_, rt, 2 h, (*S*)-**55** 91%, (*R*)-**55** 92%, (*S*)-**56** 82%, (*R*)-**56 82**%, (*S*)-**57** 89%, (*R*)-**57** 96%, (**e**) CF_3_COOH, CH_2_Cl_2_, rt, 4 h, (*S*)-**58** 98%, (*R*)-**58** 92%, (*S*)-**59** quant., (*R*)-**59** 89%, (*S*)-**60** quant., (*R*)-**60** quant.

Each trifluoroacetyl-substituted lactisole *tert*-butyl esters (**37**, **38**, **45**) was subjected to diazirine constructions, oximation with hydroxylamine hydrochloride **46**–**48**, tosylation for hydroxyl group of oxime **49**–**51**, diaziridine formation with liquid ammonia **52**–**54**, and oxidation with activated MnO_2_ to obtain **55**–**57**, which were checked by chiral center configuration. *tert*-Butyl esters were hydrolyzed with trifluoroacetic acid to afford trifluoromethyldiazirine-based lactisole derivatives in moderate yields. The one-pot conversion of the tosyl oxime **49** to diazirine **55** [36] was also acceptable for the preparation with an identical yield (Figure 7).

### 2.2. Cell-Based Sweet Taste Assay

The synthesized photoreactive lactisole derivatives were administrated to the Flp-In 293 cell line and were expressed as inhibitors to the human sweet taste receptors, with less than 3.2 mM comprising the final concentration. (*S*)-configurations of all photoreactive lactisole derivatives were preferred for sweet taste inhibition, and the results were consistent with our previous studies for lactisole and 2,4-dichlorophenoxy propanoic acid (2,4-DP) [5]. Benzophenone photophore (**9**–**11**) is too bulky to substitute for sweet taste receptors. Azide-substituted lactisoles (**20**, **21** and **28**), which feature the linear linkage of three nitrogen atoms, had slightly higher affinity for the receptor than benzophenone derivatives. Trifluoromethyldiazirinyl substitution (**58**–**60**) had the highest affinity for sweet taste receptors among the photoreactive lactisole derivatives. The positions of photophores on aromatic rings are also influenced the activity, and the 4-position was preferred as a substitute. Fifty percent inhibitory concentration (IC_50_) values (µM) for (*S*)-**58** and (*S*)-**59** are almost identical with that of (*S*)-lactisole (Table 1, Supplementary Materials).

## 3. Discussion

The 2-phenoxypropanoic acid skeleton has a unique bioactivity and is known as a pesticide and sweetener inhibitor. It seems that the stereochemistry at the 2-position may play an important role in its biological activity. We reported a comparison of the biological activity of optically pure lactisole and 2,4-dichlorophenoxy propanoic acid, which were synthesized in an asymmetric manner. Photoaffinity labeling is one of the most reliable methods for functional analysis between small ligands and biomolecules. It is essential to prepare photophore-substituted ligand derivatives. In this paper, we achieved the asymmetric synthesis of benzophenone, azide and trifluoromethyldiazirine modified derivatives on the aromatic ring of lactisole. The Mitsunobu reaction was utilized to construct a lactisole skeleton in an asymmetric manner. Although benzophenone skeletons were acceptable for the Mitsunobu reaction conditions, azide and trifluoromethyldiazirine moieties are not suitable for the condition because of the nitrogen–nitrogen multiple bonds in their structures. The phenol derivatives of precursors for these photophores were utilized for the asymmetric synthesis of the lactisole skeleton, followed by the construction of photophores on these derivatives. Several instabilities of the intermediates to the reaction condition were found, which were overcome with improvements of the reaction condition or protecting groups. Human sweet receptor assays for the photophore-containing lactisole derivatives revealed that (*S*)-isomers at the 4-position of lactisole derivatives are important and that the trifluoromethyldiazirine-substituted derivatives have identical affinity to optically pure lactisole. These results indicated that photoaffinity labeling with synthetic lactisole derivatives will be useful for the functional analysis of sweet taste receptors.

## 4. Materials and Methods

### 4.1. General Procedures

All reagents used were of analytical grade. Nuclear magnetic resonance (NMR) spectra were measured by an EX 270 spectrometer (JEOL, Tokyo, Japan). Optical rotations were measured at 23 °C on a JASCO DIP370 polarimeter (JASCO, Tokyo, Japan). HRMS-ESI spectra were obtained with a Waters (Waters, Milford, CT, USA). Chiral lactisoles ((*S*)- and (*R*)-**61**) and 2,4-DPs ((*S*)- and (*R*)-**62**) were synthesized previously [5]. The chirality of ester derivatives was determined by a Chiralcel IG column (250 × 4.6 mm) with *n*-hexane and 2-propanol.

### 4.2. Synthesis of Benzophenone-Based Lactisole Derivatives

**Methyl (*S*)-2-(4-benzoylphenoxy)propanoate ((*S*)-6).** To 4-hydroxybenzophenone **1** (182 mg, 0.92 mmol) in dry CH_2_Cl_2_ (6 mL), methyl D-(+)-lactate **4** (144 mg, 1.38 mmol) and PPh_3_ (290 mg, 1.10 mmol) was added at 0 °C. After the reaction mixture was stirred for 10 min at 0 °C, DEAD (240 mg, 1.38 mmol) was slowly added at same temperature. The reaction mixture was stirred overnight at room temperature and partitioned between water and CH_2_Cl_2_. The organic layer was washed with brine, dried over MgSO_4_, filtered and concentrated. The residue was purified by column chromatography (ethyl acetate/*n*-hexane, 1:9) to give methyl (*S*)-2-(4-benzoylphenoxy)propanoate ((*S*)-**6**) (230 mg, 88%). [α]_D_ −42 (*c* 1, CHCl_3_). ^1^H-NMR (270 MHz, CDCl_3_): *δ* = 7.81 (d, *J* = 8.6 Hz, 2H), 7.75 (d, *J* = 7.4 Hz, 2H), 7.56 (t, *J* = 7.4 Hz, 1H), 7.46 (t, *J* = 7.4 Hz, 2H), 6.93 (d, *J* = 8.6 Hz, 2H), 4.87 (q, *J* = 6.8 Hz, 1H), 3.78 (s, 3H), 1.67 (d, *J* = 6.8 Hz, 3H). ^13^C-NMR (67.5 MHz, CDCl_3_): *δ* = 195.4, 171.9, 161.0, 138.0, 132.5, 131.9, 130.9, 129.7, 128.1, 114.4, 72.4, 52.4, 18.3. HRMS (ESI): *m*/*z* calculated for C_17_H_16_O_4_ + H^+^ [M + H^+^]: 285.1127. Found: 285.1120. Chiral HPLC (*n*-hexane/2-propanol 90:10): *t*_R_ 25.8 min.

**Methyl (*R*)-2-(4-benzoylphenoxy)propanoate ((*R*)-6).** The similar treatment of 4-hydroxybenzophenone **1** (361 mg, 1.82 mmol) and methyl L-(−)-lactate **5** (284 mg, 2.72 mmol) as that just described gave (*R*)-**6** (542 mg, quant). [α]_D_ +42 (*c* 1, CHCl_3_). ^1^H-NMR (270 MHz, CDCl_3_): *δ* = 7.80 (d, *J* = 8.9 Hz, 2H), 7.74 (d, *J* = 7.3 Hz, 2H), 7.55 (t, *J* = 7.3 Hz, 1H), 7.46 (t, *J* = 7.3 Hz, 2H), 7.46 (t, *J* = 7.3 Hz, 2H), 6.92 (d, *J* = 8.9 Hz, 2H), 4.87 (q, *J* = 6.8 Hz, 1H), 3.77 (s, 3H), 1.66 (d, *J* = 6.8 Hz, 3H). ^13^C-NMR (67.5 MHz, CDCl_3_): *δ* = 195.2, 171.9, 160.9, 138.0, 132.4, 131.9, 130.8, 129.6, 128.2, 114.3, 72.3, 52.3, 18.3. HRMS (ESI): *m*/*z* calculated for C_17_H_16_O_4_ + H^+^ [M + H^+^]: 285.1127. Found: 285.1132. Chiral HPLC (*n*-hexane/2-propanol 90:10): *t*_R_ 28.0 min.

**Methyl (*S*)-2-(3-benzoylphenoxy)propanoate ((*S*)-7).** The similar treatment of 3-hydroxybenzophenone **2** (200 mg, 1.01 mmol) and methyl D-(+)-lactate **4** (157 mg, 1.51 mmol) as that just described gave (*S*)-**7** (288 mg, quant). [α]_D_ −28 (*c* 1, CHCl_3_). ^1^H-NMR (270 MHz, CDCl_3_): *δ* = 7.79 (d, *J* = 7.4 Hz, 2H), 7.59 (t, *J* = 7.4 Hz, 1H), 7.47 (t, *J* = 7.4 Hz, 2H), 7.38 (m, 2H), 7.30 (m, 1H), 7.12 (m, 1H), 4.83 (q, *J* = 6.8 Hz, 1H), 3.76 (s, 3H), 1.64 (d, *J* = 6.8 Hz, 3H). ^13^C-NMR (67.5 MHz, CDCl_3_): *δ* = 196.1, 172.2, 157.4, 138.9, 137.4, 132.4, 130.0, 129.4, 128.2, 123.5, 119.6, 115.8, 72.5, 52.3, 18.4. HRMS (ESI): *m*/*z* calculated for C_17_H_16_O_4_ + H^+^ [M + H^+^]: 285.1127. Found: 285.1127. Chiral HPLC (*n*-hexane/2-propanol 90:10): *t*_R_ 19.6 min.

**Methyl (*R*)-2-(3-benzoylphenoxy)propanoate ((*R*)-7).** The similar treatment of 3-hydroxybenzophenone **2** (360 mg, 1.82 mmol) and methyl L-(−)-lactate **5** (284 mg, 2.72 mmol) as that just described gave (*R*)-**7** (517 mg, quant). [α]_D_ +28 (*c* 1, CHCl_3_). ^1^H-NMR (270 MHz, CDCl_3_): *δ* = 7.71 (d, *J* = 7.4 Hz, 2H), 7.51 (t, *J* = 7.4 Hz, 1H), 7.39 (t, *J* = 7.4 Hz, 2H), 7.31 (m, 2H), 7.22 (m, 1H), 7.04 (m, 1H), 4.75 (q, *J* = 6.8 Hz, 1H), 3.68 (s, 3H), 1.56 (d, *J* = 6.8 Hz, 3H). ^13^C-NMR (67.5 MHz, CDCl_3_): *δ* = 196.1, 172.2 157.4, 138.9, 137.4, 132.4, 129.7, 128.2, 123.5, 119.6, 115.9, 72.6, 52.3, 18.5. HRMS (ESI): *m*/*z* calculated for C_17_H_16_O_4_ + H^+^ [M + H^+^]: 285.1127. Found: 285.1122. Chiral HPLC (*n*-hexane/2-propanol 90:10): *t*_R_ 18.9 min.

**Methyl (*S*)-2-(2-benzoylphenoxy)propanoate ((*S*)-8).** The similar treatment of 2-hydroxybenzophenone **3** (210 mg, 1.06 mmol) and methyl D-(+)-lactate **4** (165 mg, 1.59 mmol) as that just described gave (*S*)-**8** (263 mg, 88%). [α]_D_ +11 (*c* 1, CHCl_3_). ^1^H-NMR (270 MHz, CDCl_3_): *δ* = 7.84 (2H, d, *J* = 8.2 Hz), 7.54 (1H, t, *J* = 7.4 Hz), 7.43 (1H, d, *J* = 7.6 Hz), 7.42 (3H, t, *J* = 7.4 Hz), 7.08 (1H, t, *J* = 7.4 Hz), 6.80 (1H, d, *J* = 8.2 Hz), 4.66 (1H, q, *J* = 6.8 Hz), 3.68 (3H, s), 1.24 (3H, d, *J* = 6.8 Hz). ^13^C-NMR (67.5 MHz, CDCl_3_): *δ* = 196.3, 172.0, 155.3, 138.0, 132.7, 131.9, 130.0, 129.7, 129.7, 128.0, 121.6, 113.0, 73.2, 52.2, 18.0. HRMS (ESI): *m*/*z* calculated for C_17_H_16_O_4_ + H^+^ [M + H^+^]: 285.1127. Found: 285.1119. Chiral HPLC (*n*-hexane:2-propanol = 90:10) *t*_R_ 15.4 min.

**Methyl (*R*)-2-(2-benzoylphenoxy)propanoate ((*R*)-8).** The similar treatment of 2-hydroxybenzophenone **3** (372 mg, 1.87 mmol) and methyl L-(−)-lactate **5** (293 mg, 2.81 mmol) as that just described gave (*R*)-**8** (435 mg, 82%). [α]_D_ −11 (*c* 1, CHCl_3_). ^1^H-NMR (270 MHz, CDCl_3_): *δ* = 7.83 (d, *J* = 8.2 Hz, 2H), 7.54 (t, *J* = 7.4 Hz, 1H), 7.43 (d, *J* = 7.6 Hz, 1H), 7.42 (t, *J* = 7.9 Hz, 3H), 7.08 (t, *J* = 7.4 Hz, 1H), 6.80 (d, *J* = 8.2 Hz, 1H), 4.66 (q, *J* = 6.8 Hz, 1H), 3.68 (s, 3H), 1.24 (d, *J* = 6.8 Hz, 3H). ^13^C-NMR (67.5 MHz, CDCl_3_): *δ* = 196.3, 172.0, 155.3, 138.0, 132.7, 131.8, 130.0, 129.7, 129.7, 128.0, 121.6, 113.0, 73.2, 52.1, 17.9. HRMS (ESI): *m*/*z* calculated for C_17_H_16_O_4_ + H^+^ [M + H^+^]: 285.1127. Found: 285.1132. Chiral HPLC (*n*-hexane:2-propanol = 90:10) *t*_R_ 17.7 min.

**(*S*)-2-(4-benzoylphenoxy)propanoic acid ((*S*)-9).** Methyl (*S*)-2-(4-benzoylphenoxy)-propanoate (*S*)-**6** (212 mg, 0.75 mmol) was dissolved in MeOH (14.4 mL) and H_2_O (1.6 mL), and then K_2_CO_3_ (309 mg, 2.24 mmol) was added. After the reaction mixture was stirred at reflux for 2 h, cooled to room temperature and then partitioned between ethyl acetate and water. The water layer was acidified by 1 M HCl and extracted by ethyl acetate. The organic layer was washed by H_2_O and brine, and dried over MgSO_4_, filtrated and concentrated to give (*S*)-2-(2-benzoylphenoxy)propanoic acid (*S*)-**9** (206 mg, quant). [α]_D_ −22 (*c* 1, CHCl_3_). ^1^H-NMR (270 MHz, CDCl_3_): *δ* = 7.82 (d, *J* = 8.9 Hz, 2H), 7.75 (d, *J* = 7.4 Hz, 2H), 7.57 (t, *J* = 7.4 Hz, 1H), 7.47 (t, *J* = 7.4 Hz, 2H), 6.95 (d, *J* = 8.9 Hz, 2H), 4.91 (q, *J* = 6.8 Hz, 1H), 1.72 (d, *J* = 6.8 Hz, 3H). ^13^C-NMR (67.5 MHz, CDCl_3_): *δ* = 195.9, 176.4, 160.9, 137.8, 132.6, 132.1, 130.8, 129.8, 128.2, 114.4, 71.9, 18.3. HRMS (ESI): *m*/*z* calculated for C_16_H_14_O_4_ + H^+^ [M + H^+^]: 271.0970. Found: 271.0970.

**(*R*)-2-(4-benzoylphenoxy)propanoic acid ((*R*)-9).** The similar treatment of methyl (*R*)-2-(4-benzoylphenoxy)propanoate (*R*)-**6** (517 mg, 1.82 mmol) as that just described gave (*R*)-**9** (511 mg, quant). [α]_D_ +22 (*c* 1, CHCl_3_). ^1^H-NMR (270 MHz, CDCl_3_): *δ* = 7.82 (d, *J* = 8.9 Hz, 2H), 7.75 (d, *J* = 7.4 Hz, 2H), 7.57 (t, *J* = 7.4 Hz, 1H), 7.47 (t, *J* = 7.4 Hz, 2H), 6.96 (d, *J* = 8.9 Hz, 2H), 4.91 (q, *J* = 6.8 Hz, 1H), 1.72 (d, *J* = 6.8 Hz, 3H). ^13^C-NMR (67.5 MHz, CDCl_3_): *δ* = 196.0, 175.8, 161.0, 137.8, 132.6, 132.1, 130.8, 129.8, 128.2, 114.5, 72.0, 18.3. HRMS (ESI): *m*/*z* calculated for C_16_H_14_O_4_ + H^+^ [M + H^+^]: 271.0970. Found: 271.0950.

**(*S*)-2-(3-benzoylphenoxy)propanoic acid ((*S*)-10).** The similar treatment of methyl (*S*)-2-(3-benzoylphenoxy)propanoate (*S*)-**7** (159 mg, 0.56 mmol) as that just described gave (*S*)-**10** (144 mg, 95%). [α]_D_ −9 (*c* 1, CHCl_3_). ^1^H-NMR (270 MHz, CDCl_3_): *δ* = 7.78 (d, *J* = 7.3 Hz, 2H), 7.57 (t, *J* = 7.3 Hz, 1H), 7.46 (t, *J* = 7.3 Hz, 2H), 7.40 (m, 2H), 7.3 (m, 1H), 7.15 (m, 1H), 4.88 (q, *J* = 6.8 Hz, 1H), 1.68 (d, *J* = 6.8 Hz, 3H). ^13^C-NMR (67.5 MHz, CDCl_3_): *δ* = 196.3, 177.0, 157.2, 139.0, 137.3, 132.6, 130.0, 129.6, 128.3, 123.9, 119.7, 116.0, 72.1, 18.3. HRMS (ESI): *m*/*z* calculated for C_16_H_14_O_4_ + H^+^ [M + H^+^]: 271.0970. Found: 271.0952. Chiral HPLC (*n*-hexane:2-propanol = 90:10) *t*_R_ 25.8 min.

**(*R*)-2-(3-benzoylphenoxy)propanoic acid ((*R*)-10).** The similar treatment of methyl (*R*)-2-(3-benzoylphenoxy)propanoate (*R*)-**7** (413 mg, 1.45 mmol) as that just described gave (*R*)-**10** (465 mg, quant). [α]_D_ +9 (*c* 1, CHCl_3_). ^1^H-NMR (270 MHz, CDCl_3_): *δ* = 7.78 (d, *J* = 7.4 Hz, 2H), 7.57 (t, *J* = 7.4 Hz, 1H), 7.45 (t, *J* = 7.4 Hz, 2H), 7.39 (m, 2H), 7.33 (m, 1H), 7.14 (m, 1H), 4.88 (q, *J* = 6.8 Hz, 1H), 1.68 (d, *J* = 6.8 Hz, 3H). ^13^C-NMR (67.5 MHz, CDCl_3_): *δ* = 196.5, 176.5, 157.2, 138.8, 137.2, 132.6, 130.0, 129.5, 128.2, 123.8, 119.7, 115.9, 72.1, 18.3. HRMS (ESI): *m*/*z* calculated for C_16_H_14_O_4_ + H^+^ [M + H^+^]: 271.0970. Found: 271.0960. Chiral HPLC (*n*-hexane:2-propanol = 90:10) *t*_R_ 28.0 min.

**(*S*)-2-(2-benzoylphenoxy)propanoic acid ((*S*)-11).** The similar treatment of methyl (*S*)-2-(3-benzoylphenoxy)propanoate (*S*)-**8** (229 mg, 0.80 mmol) as that just described gave (*S*)-**11** (187 mg, 87%). [α]_D_ −40 (*c* 1, CHCl_3_). ^1^H-NMR (270 MHz, CDCl_3_): *δ* = 7.87 (d, *J* = 7.3 Hz, 2H), 7.64 (t, *J* = 7.3 Hz, 1H), 7.50 (m, 4H), 7.12 (m, 2H), 5.00 (q, *J* = 6.8 Hz, 1H), 1.65 (d, *J* = 6.8 Hz, 3H). ^13^C-NMR (67.5 MHz, CDCl_3_): *δ* = 197.1, 173.1, 156.6, 137.0, 133.8, 132.1, 130.7, 128.5, 127.3, 121.9, 115.1, 75.2, 18.6. HRMS (ESI): *m*/*z* calculated for C_16_H_14_O_4_ + H^+^ [M + H^+^]: 271.0970. Found: 271.0950.

**(*R*)-2-(2-benzoylphenoxy)propanoic acid ((*R*)-11).** The similar treatment of methyl (*S*)-2-(2-benzoylphenoxy)propanoate (*R*)-**8** (392 mg, 1.38 mmol) as that just described gave (*R*)-**11** (326 mg, 88%). [α]_D_ +40 (*c* 1, CHCl_3_). ^1^H-NMR (270 MHz, CDCl_3_): *δ* = 7.87 (d, *J* = 6.9 Hz, 2H), 7.64 (t, *J* = 7.4 Hz, 1H), 7.50 (m, 4H), 7.12(m, 2H), 5.01 (q, *J* = 6.9 Hz, 1H), 1.66 (d, *J* = 6.9 Hz, 3H). ^13^C-NMR (67.5 MHz, CDCl_3_): *δ* = 197.1, 173.0, 156.6, 137.0, 133.8, 132.1, 130.7, 128.5, 127.3, 121.9, 115.0, 75.2, 18.6. HRMS (ESI): *m*/*z* calculated for C_16_H_14_O_4_ + H^+^ [M + H^+^]: 271.0970. Found: 271.0950.

### 4.3. Synthesis of 3- or 4-Azidephenoxy-Based Lactisole Derivatives

**Methyl (*S*)-2-(4-nitrophenoxy)propanoate ((*S*)-14)**. To 4-nitrophenol **12** (437 mg, 3.14 mmol) in dry CH_2_Cl_2_ (6 mL), D-(+)-lactate **4** (491 mg, 4.71 mmol) and PPh_3_ (989 mg, 3.77 mmol) was added at 0 °C After the reaction mixture was stirred for 10 min at 0 °C, DEAD (820 mg, 4.71 mmol) was slowly added at same temperature. The reaction mixture was stirred overnight at room temperature and partitioned between water and CH_2_Cl_2_. The organic layer was washed with brine, dried over MgSO_4_, filtered and concentrated. The residue was purified by column chromatography (ethyl acetate/*n*-hexane, 1:9) to give (*S*)-**14** (700 mg, 99%). [α]_D_ −64 (*c* 1, CHCl_3_). ^1^H-NMR (270 MHz, CDCl_3_): *δ* = 8.20 (d, *J* = 8.9 Hz, 2H), 6.93 (d, *J* = 8.9 Hz, 2H), 4.88 (q, *J* = 6.8 Hz, 1H), 3.78 (s, 3H), 1.68 (d, *J* = 6.8 Hz, 3H). ^13^C-NMR (67.5 MHz, CDCl_3_): *δ* = 171.4, 162.4, 142.0, 125.9, 114.9, 72.9, 52.6, 18.4. HRMS (ESI): *m*/*z* calculated for C_10_H_11_NO_5_ + H^+^ [M + H^+^]: 226.0715. Found: 226.0720.

**Methyl (*R*)-2-(4-nitrophenoxy)propanoate ((*R*)-14).** The similar treatment of 4-nitrophenol **12** (128 mg, 0.92 mmol) and methyl L-(−)-lactate **5** (144 mg, 1.38 mmol) as that just described gave (*R*)-**14** (213 mg, quant). [α]_D_ +64 (*c* 1, CHCl_3_), (ref. [α]_D_ +64.4 (*c* 0.10, CHCl_3_) [29]). ^1^H-NMR (270 MHz, CDCl_3_): *δ* = 8.18 (d, *J* = 8.6 Hz, 2H), 6.93 (d, *J* = 9.2 Hz, 2H), 4.90 (q, *J* = 6.8 Hz, 1H), 3.78 (s, 3H), 1.68 d, *J* = 6.8 Hz, 3H). ^13^C-NMR (67.5 MHz, CDCl_3_): *δ* = 171.2, 162.3, 141.9, 125.8, 114.8, 72.7, 52.5, 18.2. HRMS (ESI): *m*/*z* calculated for C_10_H_11_NO_5_ + H^+^ [M + H^+^]: 226.0715. Found: 226.0720.

**Methyl (*S*)-2-(3-nitrophenoxy)propanoate ((*S*)-15).** The similar treatment of 3-nitrophenol **13** (301 mg, 2.16 mmol) and methyl D-(+)-lactate **4** (338 mg, 3.24 mmol) as that just described gave (*S*)-**15** (490 mg, quant). [α]_D_ −59 (*c* 1, CHCl_3_). ^1^H-NMR (270 MHz, CDCl_3_): *δ* = 7.85 (dd, *J* = 8.1, 2.3 Hz, 1H), 7.69 (t, *J* = 2.3 Hz, 1H), 7.44 (t, *J* = 8.1 Hz, 1H), 7.22 (dd, *J* = 8.1, 2.3 Hz, 1H), 4.87 (q, *J* = 6.8 Hz, 1H), 3.79 (s, 3H), 1.67 (d, *J* = 6.8 Hz, 3H). ^13^C-NMR (67.5 MHz, CDCl_3_): *δ* = 171.5, 158.0, 149.1, 130.1, 121.8, 116.5, 109.7, 72.9, 52.5, 18.3. HRMS (ESI): *m*/*z* calculated for C_10_H_11_NO_5_ + H^+^ [M + H^+^]: 226.0715. Found: 226.0730.

**Methyl (*R*)-2-(3-nitrophenoxy)propanoate ((*R*)-15).** The similar treatment of 3-nitrophenol **13** (422 mg, 3.04 mmol) and methyl L-(−)-lactate **5** (474 mg, 4.55 mmol) as that just described gave (*R*)-**15** (711 mg, quant). [α]_D_ +59 (*c* 1, CHCl_3_). ^1^H-NMR (270 MHz, CDCl_3_): *δ* = 7.85 (dd, *J* = 8.2, 2.3 Hz, 1H), 7.69 (t, *J* = 2.3 Hz, 1H), 7.44 (t, *J* = 8.2 Hz, 1H), 7.22 (dd, *J* = 8.2, 2.3 Hz, 1H), 4.87 (q, *J* = 6.8 Hz, 1H), 3.79 (s, 3H), 1.67 (d, *J* = 6.8 Hz, 3H). ^13^C-NMR (67.5 MHz, CDCl_3_): *δ* = 171.5, 158.0, 149.1, 130.1, 121.8, 116.5, 109.7, 72.9, 52.5, 18.3. HRMS (ESI): *m*/*z* calculated for C_10_H_11_NO_5_ + H^+^ [M + H^+^]: 226.0715. Found: 226.0710.

**Methyl (*S*)-2-(4-aminophenoxy)propanoate ((*S*)-16).** Methyl (*S*)-2-(4-nitrophenoxy)-propanoate (*S*)-**15** (700 mg, 3.11 mmol) and 5% Pd/C (35.0 mg) were suspended in MeOH (10 mL). The reaction mixture was stirred vigorously at room temperature for 2 h under H_2_ atmosphere. The residue was filtrated using Celite and concentrated to give (*S*)-**16** (607 mg, quant). [α]_D_ −50 (*c* 1, CHCl_3_). ^1^H-NMR (270 MHz, CDCl_3_): *δ* = 6.73 (d, *J* = 8.9 Hz, 2H), 6.59 (d, *J* = 8.9 Hz, 2H), 4.63 (q, *J* = 6.8 Hz, 1H), 3.73 (s, 3H), 3.46 (s, 2H), 1.57 (d, *J* = 6.8 Hz, 3H). ^13^C-NMR (67.5 MHz, CDCl_3_): *δ* = 173.0, 150.4, 141.0, 116.7, 116.1, 73.7, 52.1, 18.5. HRMS (ESI): *m*/*z* calculated for C_10_H_13_NO_3_ + H^+^ [M + H^+^]: 196.0974. Found: 196.0960.

**Methyl (*R*)-2-(4-aminophenoxy)propanoate ((*R*)-16).** The similar treatment of methyl (*R*)-2-(4-nitrophenoxy)propanoate (*R*)-**15** (575 mg, 2.56 mmol) as that just described gave (*R*)-**16** (500 mg, quant). [α]_D_ +50 (*c* 1, CHCl_3_). ^1^H-NMR (270 MHz, CDCl_3_): *δ* = 6.73 (d, *J* = 8.9 Hz, 2H), 6.66 (d, *J* = 8.9 Hz, 2H), 4.64 (q, *J* = 6.8 Hz, 1H), 4.24 (s, 2H), 3.73 (s, 3H), 1.57 (3H, d, *J* = 6.8 Hz, 3H). ^13^C-NMR (67.5 MHz, CDCl_3_): *δ* = 172.9, 151.0, 139.5, 116.9, 116.7, 73.6, 52.1, 18.5. HRMS (ESI): *m*/*z* calculated for C_10_H_13_NO_3_ + H^+^ [M + H^+^]: 196.0974. Found: 196.0950.

**Methyl (*S*)-2-(3-aminophenoxy)propanoate ((*S*)-17).** The similar treatment of methyl (*S*)-2-(3-nitrophenoxy)propanoate (*S*)-**15** (490 mg, 2.17 mmol) as that just described gave (*S*)-**17** (367 mg, 87%). [α]_D_ −23 (*c* 1, CHCl_3_). ^1^H-NMR (270 MHz, CDCl_3_): *δ* = 7.01 (t, *J* = 7.9 Hz, 1H), 6.28 (dd, *J* = 7.9, 1.0 Hz, 1H), 6.23 (m, 2H), 4.72 (q, *J* = 6.8 Hz, 1H), 3.73 (s, 3H), 3.68 (s, 3H), 1.58 (d, *J* = 6.8 Hz, 3H). ^13^C-NMR (67.5 MHz, CDCl_3_): *δ* = 172.7, 158.5, 147.9, 130.0, 108.7, 104.4, 102.1, 72.2, 52.1, 18.5. HRMS (ESI): *m*/*z* calculated for C_10_H_13_NO_3_ + H^+^ [M + H^+^]: 196.0974. Found: 196.0980.

**Methyl (*R*)-2-(3-aminophenoxy)propanoate ((*R*)-17).** The similar treatment of methyl (*R*)-2-(3-nitrophenoxy)propanoate (*R*)-**15** (628 mg, 2.78 mmol) as that just described gave (*R*)-**17** (558 mg, quant). [α]_D_ +23 (*c* 1, CHCl_3_). ^1^H-NMR (270 MHz, CDCl_3_): *δ* = 7.05 (t, *J* = 7.9 Hz, 1H), 6.37 (d, *J* = 7.9 Hz, 1H), 6.25 (m, 2H), 4.73 (q, *J* = 6.8 Hz, 1H), 4.19 (s, 2H), 3.75 (s, 3H), 1.59 (d, *J* = 6.8 Hz, 3H). ^13^C-NMR (67.5 MHz, CDCl_3_): *δ* = 172.8, 158.6, 146.7, 130.2, 109.3, 105.3, 102.9, 72.4, 52.3, 18.5. HRMS (ESI): *m*/*z* calculated for C_10_H_13_NO_3_ + H^+^ [M + H^+^]: 196.0974. Found: 196.0960.

**Methyl (*S*)-2-(4-azidephenoxy)propanoate ((*S*)-18).** Methyl (*S*)-2-(4-aminophenoxy)-propanoate (*S*)-**14** (607 mg, 3.11 mmol) was dissolved in water (6.78 mL) and 37% HCl (0.76 mL) at 0 °C. NaNO_2_ (237 mg, 3.44 mmol) in water (2.26 mL) was added slowly at 0 °C, followed by NaN_3_ (381 mg, 5.87 mmol) in water (2.26 mL). After 30 min at 0 °C and 30 min at room temperature, the reaction mixture was extracted with ethyl acetate. The organic layer was washed by brine, dried over MgSO_4_, filtrated and concentrated to give (*S*)-**18** (702 mg, quant). [α]_D_ −45 (*c* 1, CHCl_3_). ^1^H-NMR (270 MHz, CDCl_3_): *δ* = 6.94 (d, *J* = 9.2 Hz, 2H), 6.86 (d, *J* = 9.2 Hz, 2H), 4.72 (q, *J* = 6.8 Hz, 1H), 3.76 (s, 3H), 1.61 (d, *J* = 6.8 Hz, 3H). ^13^C-NMR (67.5 MHz, CDCl_3_): *δ* = 172.4, 154.9, 133.4, 120.1, 116.5, 73.0, 52.3, 18.5. HRMS (ESI): *m*/*z* calculated for C_10_H_11_N_3_O_3_ + H^+^ [M + H^+^]: 222.0879. Found: 222.0860. Chiral HPLC (*n*-hexane/2-propanol 99:1): *t*_R_ 20.5 min.

**Methyl (*R*)-2-(4-azidephenoxy)propanoate ((*R*)-18).** The similar treatment of methyl (*R*)-2-(4-aminophenoxy)propanoate (*R*)-**14** (500 mg, 2.56 mmol) as that just described gave (*R*)-**18** (529 mg, 93%). [α]_D_ +45 (*c* 1, CHCl_3_). ^1^H-NMR (270 MHz, CDCl_3_): *δ* = 6.94 (d, *J* = 9.2 Hz, 2H), 6.86 (d, *J* = 9.2 Hz, 2H), 4.73 (q, *J* = 6.8 Hz, 1H), 3.76 (s, 3H), 1.61 (d, *J* = 6.8 Hz, 3H). ^13^C-NMR (67.5 MHz, CDCl_3_): *δ* = 172.4, 154.9, 133.3, 120.1, 116.5, 73.0, 52.3, 18.5. HRMS (ESI): *m*/*z* calculated for C_10_H_11_N_3_O_3_ + H^+^ [M + H^+^]: 222.0879. Found: 222.0870. Chiral HPLC (*n*-hexane/2-propanol 99:1): *t*_R_ 19.4 min.

**Methyl (*S*)-2-(3-azidophenoxy)propanoate ((*S*)-19).** The similar treatment of methyl (*S*)-2-(3-aminophenoxy)propanoate (*S*)-**17** (323 mg, 1.65 mmol) as that just described gave (*S*)-**19** (336 mg, 92%). [α]_D_ −28 (*c* 1, CHCl_3_). ^1^H-NMR (270 MHz, CDCl_3_): *δ* = 7.24 (t, *J* = 8.2 Hz, 1H), 6.65 (t, *J* = 8.2 Hz, 2H), 6.55 (s, 1H), 4.76 (q, *J* = 6.8 Hz, 1H), 3.76 (s, 3H), 1.62 (d, *J* = 6.8 Hz, 3H). ^13^C-NMR (67.5 MHz, CDCl_3_): *δ* = 172.1, 158.6, 141.3, 130.5, 112.2, 111.1, 106.3, 72.5, 52.3, 18.4. HRMS (ESI): *m*/*z* calculated for C_10_H_11_N_3_O_3_ + H^+^ [M + H^+^]: 222.0879. Found: 222.0880. Chiral HPLC (*n*-hexane/2-propanol 99:1): *t*_R_ 18.0 min.

**Methyl (*R*)-2-(3-azidophenoxy)propanoate ((*R*)-19).** The similar treatment of methyl (*R*)-2-(3-aminophenoxy)propanoate (*R*)-**17** (518 mg, 2.65 mmol) as that just described gave (*R*)-**19** (497 mg, 85%). [α]_D_ +28 (*c* 1, CHCl_3_). ^1^H-NMR (270 MHz, CDCl_3_): *δ* = 7.24 (t, *J* = 8.1 Hz, 1H), 6.65 (t, *J* = 8.1 Hz, 1H), 6.55 (s, 1H), 4.76 (q, *J* = 6.8 Hz, 1H), 3.76 (s, 3H), 1.62 (d, *J* = 6.8 Hz, 3H). ^13^C-NMR (67.5 MHz, CDCl_3_): *δ* = 172.0, 158.5, 141.2, 130.4, 112.0, 111.0, 106.2, 72.3, 52.1, 18.2. HRMS (ESI): *m*/*z* calculated for C_10_H_11_N_3_O_3_ + H^+^ [M + H^+^]: 222.0879. Found: 222.0860. Chiral HPLC (*n*-hexane/2-propanol 99:1): *t*_R_ 16.7 min.

**(*S*)-2-(4-Azidophenoxy)propanoic acid ((*S*)-20).** Methyl (*S*)-2-(4-azidophenoxy)propanoate (*S*)-**18** (702 mg, 3.17 mmol) was dissolved in MeOH (15 mL) and 2M NaOH (3.2 mL). After the reaction mixture was stirred at reflux for 2 h, cooled to room temperature and then partitioned between ethyl acetate and water. The water layer was acidified by 1 M HCl aq and extracted by ethyl acetate. The organic layer was washed by H_2_O and brine, and dried over MgSO_4_, filtrated and concentrated to give (*S*)-**20** (601 mg, 91%). [α]_D_ +27 (*c* 1, CHCl_3_). ^1^H-NMR (270 MHz, CDCl_3_): *δ* = 9.68 (s, 1H), 6.96 (d, *J* = 8.9 Hz, 4H), 6.89 (d, *J* = 8.9 Hz, 4H), 4.75 (d, *J* = 6.9 Hz, 1H), 1.66 (d, *J* = 6.9 Hz, 3H). ^13^C-NMR (67.5 MHz, CDCl_3_): *δ* = 177.6, 154.5, 133.7, 120.2, 116.6, 72.5, 18.4. HRMS (ESI): *m*/*z* calculated for C_9_H_9_N_3_O_3_ + H^+^ [M + H^+^]: 208.0722. Found: 208.0720.

**(*R*)-2-(4-Azidophenoxy)propanoic acid ((*R*)-20).** The similar treatment of methyl (*R*)-2-(4-azidophenoxy)propanoate (*R*)-**18** (529 mg, 2.39 mmol) as that just described gave (*R*)-**20** (475 mg, 96%). [α]_D_ −27 (*c* 1, CHCl_3_). ^1^H-NMR (270 MHz, CDCl_3_): *δ* = 11.71 (s, 1H), 6.93 (d, *J* = 8.9 Hz, 2H), 6.87 (d, *J* = 8.9 Hz, 2H), 4.74 (q, *J* = 6.8 Hz, 1H), 1.65 (d, *J* = 6.8 Hz, 3H). ^13^C-NMR (67.5 MHz, CDCl_3_): *δ* = 178.1, 154.5, 133.7, 120.1, 116.6, 72.5, 18.4. HRMS (ESI): *m*/*z* calculated for C_9_H_9_N_3_O_3_ + H^+^ [M + H^+^]: 208.0722. Found: 208.0710.

**(*S*)-2-(3-Azidophenoxy)propanoic acid ((*S*)-21).** The similar treatment of methyl (*S*)-2-(3-azidephenoxy)propanoate (*S*)-**19** (212 mg, 0.96 mmol) as that just described gave (*S*)-**21** (211 mg, quant). [α]_D_ −5 (*c* 1, CHCl_3_). ^1^H-NMR (270 MHz, CDCl_3_): *δ* = 7.26 (t, *J* = 8.1 Hz, 1H), 6.70 (dd, *J* = 8.1, 2.1 Hz, 1H), 6.66 (dd, *J* = 8.1, 2.1 Hz, 1H), 6.57 (t, *J* = 2.1 Hz, 1H), 4.80 (q, *J* = 6.8 Hz, 1H), 1.67 (d, *J* = 6.6 Hz, 3H). ^13^C-NMR (67.5 MHz, CDCl_3_): *δ* = 177.5, 158.4, 141.6, 130.7, 112.5, 111.2, 106.5, 72.0, 18.3. HRMS (ESI): *m*/*z* calculated for C_9_H_9_N_3_O_3_ + H^+^ [M + H^+^]: 208.0722. Found: 208.0750.

**(*R*)-2-(3-Azidophenoxy)propanoic acid ((*R*)-21)**. The similar treatment of methyl (*R*)-2-(3-azidophenoxy)propanoate (*R*)-**19** (386 mg, 1.74 mmol) as that just described gave (*R*)-**21** (294 mg, 81%). [α]_D_ +5 (*c* 1, CHCl_3_). ^1^H-NMR (270 MHz, CDCl_3_): *δ* = 7.25 (t, *J* = 8.1 Hz, 1H), 6.69 (dd, *J* = 8.1, 2.1 Hz, 2H), 6.65 (dd, *J* = 8.1, 2.1 Hz, 2H), 6.57 (t, *J* = 2.1 Hz, 1H), 4.79 (q, *J* = 6.8 Hz, 1H), 1.66 (d, *J* = 6.8 Hz, 3H). ^13^C-NMR (67.5 MHz, CDCl_3_): *δ* = 177.6, 158.4, 141.6, 130.7, 112.5, 111.2, 106.6, 72.0, 18.3. HRMS (ESI): *m*/*z* calculated for C_9_H_9_N_3_O_3_ + H^+^ [M + H^+^]: 208.0722. Found: 208.0710.

### 4.4. Synthesis of 2-Azidephenoxy-Based Lactisole Derivatives

**Methyl (*S*)-2-(2-nitrophenoxy)propanoate ((*S*)-23)**. The similar treatment of Mitsunobu reaction for 2-nitrophenol **22** (509 mg, 3.66 mmol) and methyl D-(+)-lactate **4** (572 mg, 5.49 mmol) as that just described gave (*S*)-**23** (952 mg, quant). [α]_D_ +106 (*c* 1, CHCl_3_). ^1^H-NMR (270 MHz, CDCl_3_): *δ* = 7.83 (dd, *J* = 8.2, 1.6 Hz, 1H), 7.49 (td, *J* = 8.2, 1.6 Hz, 1H), 7.08 (td, *J* = 8.2, 1.2 Hz, 1H), 6.96 (dd, *J* = 8.2, 1.2 Hz, 1H), 4.85 (q, *J* = 6.8 Hz, 1H), 3.76 (s, 3H), 1.69 (d, *J* = 6.8 Hz, 3H). ^13^C-NMR (67.5 MHz, CDCl_3_): *δ* = 171.1, 150.6, 140.5, 133.7, 125.4, 121.4, 115.6, 74.2, 52.3, 18.1. HRMS (ESI): *m*/*z* calculated for C_10_H_11_NO_5_ + H^+^ [M + H^+^]: 226.0715. Found: 226.0705.

**Methyl (*R*)-2-(2-nitrophenoxy)propanoate ((*R*)-23).** The similar treatment of 2-nitrophenol **22** (500 mg, 3.60 mmol) and methyl L-(−)-lactate **5** (562 mg, 5.40 mmol) as that just described gave (*R*)-**23** (892 mg, quant). [α]_D_ −106 (*c* 1, CHCl_3_). (ref. [α]_D_ −126.86 (c 1.0, EtOH) [28]). ^1^H-NMR (270 MHz, CDCl_3_): *δ* = 7.76 (dd, *J* = 8.2, 1.6 Hz, 1H), 7.41 (td, *J* = 8.2, 1.6 Hz, 1H), 7.01 (td, *J* = 8.2, 1.2 Hz, 1H), 6.89 (td, *J* = 8.2, 1.2 Hz, 1H), 4.79 (q, *J* = 6.8 Hz, 1H), 3.69 (s, 3H), 1.62 (d, *J* = 6.8 Hz, 3H). ^13^C-NMR (67.5 MHz, CDCl_3_): *δ* = 171.3, 150.8, 140.7, 133.8, 125.6, 121.5, 115.8, 74.5, 52.5, 18.3. HRMS (ESI): *m*/*z* calculated for C_10_H_11_NO_5_ + H^+^ [M + H^+^]: 226.0715. Found: 226.0710.

**(*S*)-2-(2-Nitrophenoxy)propanoic acid ((*S*)-24)**. Methyl (*S*)-2-(2-nitrophenoxy)propanoate (*S*)-**23** (261 mg, 1.16 mmol) was dissolved in MeOH (15 mL) and 2 M NaOH (1.5 mL). After the reaction mixture was stirred at reflux for 1 h, cooled to room temperature and then partitioned between ethyl acetate and water. The water layer was acidified by 1 M HCl and extracted by ethyl acetate. The organic layer was washed by H_2_O and brine, and dried over MgSO_4_, filtrated and concentrated to give (*S*)-**24** (251 mg, quant). [α]_D_ +5 (*c* 1, CHCl_3_). ^1^H-NMR (270 MHz, CDCl_3_): *δ* = 7.84 (d, *J* = 8.2 Hz, 1H), 7.49 (t, *J* = 8.2 Hz, 1H), 7.07 (t, *J* = 8.2 Hz, 1H), 6.97 (d, *J* = 8.2 Hz, 1H), 4.88 (q, *J* = 6.8 Hz, 1H), 1.68 (d, *J* = 6.8 Hz, 3H). ^13^C-NMR (67.5 MHz, CDCl_3_): *δ* = 174.4, 150.4, 140.3, 134.4, 126.1, 122.2, 115.8, 74.4, 18.2. HRMS (ESI): *m*/*z* calculated for C_9_H_9_NO_5_ + H^+^ [M + H^+^]: 212.0559. Found: 212.0540.

**(*R*)-2-(2-Nitrophenoxy)propanoic acid ((*R*)-24).** The similar treatment of methyl (*R*)-2-(2-nitrophenoxy)propanoate (*R*)-**23** (632 mg, 2.81 mmol) as that just described gave (*R*)-**24** (752 mg, quant). [α]_D_ −5 (*c* 1, CHCl_3_). ^1^H-NMR (270 MHz, CDCl_3_): *δ* = 7.79 (d, *J* = 8.2 Hz, 1H), 7.45 (t, *J* = 8.2 Hz, 1H), 7.04 (t, *J* = 8.2 Hz, 1H), 6.95 (d, *J* = 8.2 Hz, 1H), 4.85 (q, *J* = 6.8 Hz, 1H), 1.66 (d, *J* = 6.8 Hz, 3H). ^13^C-NMR (67.5 MHz, CDCl_3_): *δ* = 175.5, 150.5, 140.4, 134.3, 126.0, 122.0, 115.8, 74.1, 18.1. HRMS (ESI): *m*/*z* calculated for C_9_H_9_NO_5_ + H^+^ [M + H^+^]: 212.0559. Found: 212.0560.

***tert*-Butyl (*S*)-2-(2-nitrophenoxy)propanoate ((*S*)-25).** To a solution of (*S*)-2-(2-nitrophenoxy)propanoic acid (*S*)-**24** (251 mg, 1.19 mmol), potassium carbonate anhydride (4.11 g, 29.7 mmol) and tetrabutylammonium bromide (384 mg, 1.19 mmol) in *N*,*N*-dimethylacetamide (6 mL), *tert*-butyl bromide (7.81 g, 57.0 mmol) was added dropwise at 0 °C. The reaction mixture was stirred at 55 °C for 2.5 h. After cooling to room temperature, the mixture was poured into cold water and extracted with ethyl acetate. The organic layer was washed with H_2_O, brine, dried over MgSO_4_, filtrated and evaporated. The residue was purified by column chromatography (ethyl acetate/*n*-hexane, 1:6) to give (*S*)-**25** (246 mg, 77%). [α]_D_ +108 (*c* 1, CHCl_3_). ^1^H-NMR (270 MHz, CDCl_3_): *δ* = 7.81 (d, *J* = 8.2 Hz, 1H), 7.48 (t, *J* = 8.2 Hz, 1H), 7.05 (t, *J* = 8.2 Hz, 1H), 6.96 (d, *J* = 8.2 Hz, 1H), 4.75 (q, *J* = 6.8 Hz, 1H), 1.65 (d, *J* = 6.8 Hz, 3H), 1.42 (s, 9H). ^13^C-NMR (67.5 MHz, CDCl_3_): *δ* = 169.9, 151.0, 140.5, 133.5, 125.5, 121.0, 115.4, 82.4, 74.6, 27.7, 18.1. HRMS (ESI): *m*/*z* calculated for C_13_H_17_NO_5_ + H^+^ [M + H^+^]: 268.1185. Found: 268.1170.

***tert*-Butyl (*R*)-2-(2-nitrophenoxy)propanoate ((*R*)-25).** The similar treatment of (*R*)-2-(2-nitrophenoxy)propanoic acid (*R*)-**24** (289 mg, 1.37 mmol) as that just described gave (*R*)-**25** (301 mg, 82%). [α]_D_ −108 (*c* 1, CHCl_3_). ^1^H-NMR (270 MHz, CDCl_3_): *δ* = 7.72 (d, *J* = 8.6 Hz, 1H), 7.40 (t, *J* = 7.9 Hz, 1H), 6.97 (t, *J* = 7.9 Hz, 1H), 6.87 (d, *J* = 8.6 Hz, 1H), 4.67 (q, *J* = 6.8 Hz, 1H), 1.56 (d, *J* = 6.8 Hz, 3H), 1.33 (s, 9H). ^13^C-NMR (67.5 MHz, CDCl_3_): *δ* = 169.9, 151.0, 140.5, 133.5, 125.5, 121.0, 115.4, 82.4, 74.5, 27.7, 18.1. HRMS (ESI): *m*/*z* calculated for C_13_H_17_NO_5_ + H^+^ [M + H^+^]: 268.1185. Found: 268.1190.

***tert*-Butyl (*S*)-2-(2-aminophenoxy)propanoate ((*S*)-26).***tert*-Butyl (*S*)-2-(2-nitrophenoxy)-propanoate (*S*)-**25** (246 mg, 0.92 mmol) and 5% Pd/C (12.3 mg) were suspended in MeOH (20 mL). The reaction mixture was stirred vigorously at room temperature for 45 min under H_2_ atmosphere then was filtrated with Celite and concentrated. The residue was partitioned between ethyl acetate and 1 M HCl, and then 1 M NaOH was added to make pH 10, then extracted by ethyl acetate. The organic layer was washed by brine and dried over MgSO_4_, filtrated, and concentrated to give (*S*)-**26** (159 mg, 73%). [α]_D_ +11 (*c* 1, CHCl_3_). ^1^H-NMR (270 MHz, CDCl_3_): *δ* = 6.81 (t, *J* = 7.6 Hz, 1H), 6.73 (d, *J* = 7.6 Hz, 1H), 6.67 (m, 2H), 4.62 (q, *J* = 8 Hz, 1H), 3.83 (s, 2H), 1.60 (d, *J* = 6.8 Hz, 3H), 1.44 (s, 9H). ^13^C-NMR (67.5 MHz, CDCl_3_): *δ* = 171.5, 145.5, 137.2, 122.2, 118.1, 115.6, 113.5, 81.7, 74.0, 27.9, 18.6. HRMS (ESI): *m*/*z* calculated for C_13_H_19_NO_3_ + H^+^ [M + H^+^]: 238.1443. Found: 238.1440.

***tert*-Butyl (*R*)-2-(2-aminophenoxy)propanoate ((*R*)-26).** The similar treatment of *tert*-butyl (*R*)-2-(2-nitrophenoxy)propanoate (*R*)-**25** (666 mg, 2.49 mmol) as that just described gave (*R*)-**26** (524 mg, 89%). [α]_D_ −11 (*c* 1, CHCl_3_). ^1^H-NMR (270 MHz, CDCl_3_): *δ* = 6.80 (t, *J* = 7.6 Hz, 1H), 6.68 (m, 3H), 4.62 (q, *J* = 6.8 Hz, 1H), 3.92 (s, 2H), 1.59 (d, *J* = 6.8 Hz, 3H), 1.43 (s, 9H). ^13^C-NMR (67.5 MHz, CDCl_3_): *δ* = 171.5, 145.5, 137.2, 122.2, 118.1, 115.5, 113.5, 81.7, 74.00, 27.9, 18.6. HRMS (ESI): *m*/*z* calculated for C_13_H_19_NO_3_ + H^+^ [M + H^+^]: 238.1443. Found: 238.1430.

***tert*-Butyl (*S*)-2-(2-azidophenoxy)propanoate ((*S*)-27).***tert*-Butyl (*S*)-2-(2-aminophenoxy)-propanoate (*S*)-**26** (159 mg, 0.67 mmol) was dissolved in water (1.46 mL) and 37% hydrochloric acid (0.17 mL) at 0 °C. NaNO_2_ (51.3 mg, 0.74 mmol) in water (0.49 mL) was added slowly at 0 °C, followed by NaN_3_ (82.4 mg, 1.27 mmol) in water (0.49 mL). After 30 min at 0 °C and 30 min at room temperature, the reaction mixture was extracted with ethyl acetate. The organic layer was washed by brine, dried over MgSO_4_, filtrated and concentrated to give (*S*)-**27** (194 mg, quant). [α]_D_ +15 (*c* 1, CHCl_3_). ^1^H-NMR (270 MHz, CDCl_3_): *δ* = 6.90 (m, 3H), 6.72 (d, *J* = 7.9 Hz, 1H), 4.62 (q, *J* = 6.8 Hz, 1H), 1.55 (d, *J* = 6.8 Hz, 3H), 1.34 (s, 9H). ^13^C-NMR (67.5 MHz, CDCl_3_): *δ* = 170.6, 150.5, 128.9, 125.3, 122.1, 121.0, 114.1, 82.1, 74.1, 27.8, 18.1. HRMS (ESI): *m*/*z* calculated for C_13_H_17_N_3_O_3_ + H^+^ [M + H^+^]: 264.1348. Found: 264.1360. Chiral HPLC (*n*-hexane/2-propanol 199:1): *t*_R_ 37.8 min.

***tert*-Butyl (*R*)-2-(3-azidophenoxy)propanoate ((*R*)-27).** The similar treatment of *tert*-butyl (*R*)-2-(2-aminophenoxy)propanoate (*R*)-**26** (524 mg, 2.21 mmol) as that just described gave ((*R*)-39) (517 mg, 89%). [α]_D_ −15 (*c* 1, CHCl_3_). ^1^H-NMR (270 MHz, CDCl_3_): *δ* = 6.92 (m, 3H), 6.77 (d, *J* = 7.9 Hz, 1H), 4.67 (q, *J* = 6.8 Hz, 1H), 1.61 (d, *J* = 6.8 Hz, 3H), 1.39 (s, 9H). ^13^C-NMR (67.5 MHz, CDCl_3_): *δ* = 170.7, 150.5, 128.9, 125.3, 122.1, 121.0, 114.2, 82.1, 74.1, 27.8, 18.1. HRMS (ESI): *m*/*z* calculated for C_13_H_17_N_3_O_3_ + H^+^ [M + H^+^]: 264.1348. Found: 264.1330.

**(*S*)-2-(2-Azidophenoxy)propanoic acid ((*S*)-28).***tert*-Butyl (*S*)-2-(2-azidophenoxy)-propanoate ((*S*)-**27**) (218 mg, 0.83 mmol) was dissolved in 4 M HCl in dioxane (7.5 mL) and stirred at room temperature for 1 h, poured into 1 M NaOH solution to make alkaline. The organic layer was dried over MgSO_4_, filtrated and concentrated, and then the residue was treated with the same reaction 2 times. The aqueous layer was extracted with ethyl acetate. The organic layer was dried over MgSO_4_, filtrated and concentrated to give (*S*)-**28** (116.3 mg, 68%). [α]_D_ +12 (*c* 1, CHCl_3_). ^1^H-NMR (270 MHz, CDCl_3_): *δ* = 8,24 (s, 1H), 7.02 (m, 3H), 6.85 (d, *J* = 7.6 Hz, 1H), 4.81 (q, *J* = 6.8 Hz, 1H), 1.71 (d, *J* = 6.8 Hz, 3H). ^13^C-NMR (67.5 MHz, CDCl_3_): *δ* = 176.4, 149.4, 129.5, 125.6, 123.1, 120.8, 115.2, 73.7, 18.2. HRMS (ESI): *m*/*z* calculated for C_9_H_9_N_3_O_3_ + H^+^ [M + H^+^]: 208.0722. Found: 208.0705. Chiral HPLC (*n*-hexane/2-propanol 199:1): *t*_R_ 42.9 min.

**(*R*)-2-(2-Azidophenoxy)propanoic acid ((*R*)-28).** The similar treatment of *tert*-butyl (*R*)-2-(2-azidephenoxy)propanoate ((*R*)-**27**) (253 mg, 0.96 mmol) as that just described gave (*R*)-**28** (128.7 mg, 65%). [α]_D_ −12 (*c* 1, CHCl_3_). ^1^H-NMR (270 MHz, CDCl_3_): *δ* = 9.31 (s, 1H), 7.09–6.96 (m, 3H), 6.84 (d, *J* = 7.6 Hz, 1H), 4.81 (q, *J* = 6.8 Hz, 1H), 1.70 (d, *J* = 6.8 Hz, 3H). ^13^C-NMR (67.5 MHz, CDCl_3_): *δ* = 176.9, 149.5, 129.4, 125.5, 123.0, 120.8, 115.1, 73.6, 18.2. HRMS (ESI): *m*/*z* calculated for C_9_H_9_N_3_O_3_ + H^+^ [M + H^+^]: 208.0722. Found: 208.0715.

### 4.5. Synthesis of Trifluoromethyldiazirine-Based Lactisole Derivatives

**2,2,2-Trifluoro-1-(4-hydroxyphenyl)ethan-1-one 31.** 2,2,2-Trifluoro-1-(4-methoxyphenyl)-ethan-1-one **29** (493 mg, 2.41 mmol) and lithium chloride (950 mg, 22.4 mmol) was dissolved in dry DMF (7.5 mL). After the reaction mixture was stirred at reflux for 6 h, the mixture was cooled to room temperature and 1 M HCl was added, and then extracted with ethyl acetate. The organic layer was washed with brine and dried over MgSO_4_, filtrated and concentrated. The residue was purified by column chromatography (ethyl acetate/*n*-hexane, 1:8) to give **31** (376 mg, 82%). ^1^H-NMR (270 MHz, CDCl_3_): *δ* = 8.04 (d, *J* = 8.9 Hz, 2H), 6.96 (d, *J* = 8.9 Hz, 2H), 5.71 (s, 1H). ^13^C-NMR (67.5 MHz, CDCl_3_): *δ* = 179.0 (q, *^2^J_CF_* = 34.6 Hz), 133.1, 123.1, 116.9 (q, *^1^J_CF_* = 291.6 Hz), 116.0. HRMS (ESI): *m*/*z* calculated for C_8_H_5_F_3_O_2_ + H^+^ [M + H^+^]: 191.0320. Found: 191.0317.

**2,2,2-Trifluoro-1-(3-hydroxyphenyl)ethan-1-one 32**. 2,2,2-Trifluoro-1-(4-methoxyphenyl)-ethan-1-one **30** (1.052 g, 5.15 mmol) was dissolved in CH_2_Cl_2_ (30 mL) and cooled to −78 °C. BBr_3_ was added dropwise at −78 °C and the reaction mixture stirred at room temperature for 5 h. The mixture was cooled to 0 °C and 10% NaOH (30 mL) was added slowly, and then HCl was added to make pH 1 at same temperature. NH_4_OH was also added to make pH 7 at room temperature, then extracted by ethyl acetate. The organic layer was dried over MgSO_4_, filtrated and concentrated to give **31** (1.018 g, quant). ^1^H-NMR (270 MHz, CDCl_3_): *δ* = 7.63 (d, *J* = 7.8 Hz, 1H), 7.50 (s, 1H), 7.41 (t, *J* = 7.8 Hz, 1H), 7.18 (dd, *J* = 7.8, 2.6 Hz, 1H). ^13^C-NMR (67.5 MHz, CDCl_3_): *δ* = 180.3 (q, *^2^J_CF_* = 35.2 Hz), 156.1, 131.2, 130.5, 123.0, 122.8, 116.6 (q, *^1^J_CF_* = 291.6 Hz), 116.2. HRMS (ESI): *m*/*z* calculated for C_8_H_5_F_3_O_2_ + H^+^ [M + H^+^]: 191.0320. Found: 191.0316.

**Methyl (*S*)-2-(4-(2,2,2-trifluoroacetyl)phenoxy)propanoate (*S*)-33.** To 2,2,2-trifluoro-1-(4-hydroxyphenyl)ethanone **31** (1.175 g, 7.25 mmol) in dry CH_2_Cl_2_ (30 mL), D-(+)-lactate **4** (1.132 g, 10.9 mmol) and PPh_3_ (2.282 g, 8.70 mmol) was added at 0 °C. After the reaction mixture was stirred for 10 min at 0 °C, DEAD (1.891 g, 10.9 mmol) was slowly added at same temperature. The reaction mixture was stirred overnight at room temperature and partitioned between water and CH_2_Cl_2_. The organic layer was washed with brine, dried over MgSO_4_, filtered and concentrated. The residue was purified by column chromatography (ethyl acetate/*n*-hexane, 1:9) to give (*S*)-**33** (1.560 g, 78%). [α]_D_ −48 (*c* 1, CHCl_3_). ^1^H-NMR (270 MHz, CDCl_3_): *δ* = 7.96 (d, *J* = 8.2 Hz, 2H), 6.88 (d, *J* = 8.2 Hz, 2H), 4.82 (q, *J* = 6.8 Hz, 1H), 3.70 (s, 3H), 1.59 (d, *J* = 6.8 Hz, 3H). ^13^C-NMR (67.5 MHz, CDCl_3_): *δ* = 178.8 (q, *^2^J_CF_* = 34.6 Hz), 171.4, 163.2, 132.7, 123.4, 116.8 (q, *^1^J_CF_* = 291.4 Hz), 115.2, 72.5, 52.5, 18.3. HRMS (ESI): *m*/*z* calculated for C_12_H_11_F_3_O_4_ + H^+^ [M + H^+^]: 277.0688. Found: 277.0674.

**Methyl (*R*)-2-(4-(2,2,2-trifluoroacetyl)phenoxy)propanoate (*R*)-33.** The similar treatment of 2,2,2-trifluoro-1-(4-hydroxyphenyl)ethenone **31** (484 mg, 2.99 mmol) and methyl L-(−)-lactate **5** (466 mg, 4.48 mmol) as that just described gave (*R*)-**33** (614 mg, 74%). [α]_D_ +48 (*c* 1, CHCl_3_). ^1^H-NMR (270 MHz, CDCl_3_): *δ* = 8.04 (d, *J* = 8.2 Hz, 2H), 6.97 (d, *J* = 8.2 Hz, 2H), 4.91 (q, *J* = 6.8 Hz, 1H), 3.78 (s, 3H), 1.68 (d, *J* = 6.8 Hz, 3H). ^13^C-NMR (67.5 MHz, CDCl_3_): *δ* = 178.8 (q, *^2^J_CF_* = 34.6 Hz), 171.4, 163.2, 132.7, 123.4, 116.8 (q, *^1^J_CF_* = 291.4 Hz), 115.2, 72.5, 52.5, 18.3. HRMS (ESI): *m*/*z* calculated for C_12_H_11_F_3_O_4_ + H^+^ [M + H^+^]: 277.0688. Found: 277.0660.

**Methyl (*S*)-2-(3-(2,2,2-trifluoroacetyl)phenoxy)propanoate (*S*)-34.** The similar treatment of 2,2,2-trifluoro-1-(3-hydroxyphenyl)ethanone **32** (248 mg, 1.53 mmol) and methyl D-(−)-lactate **4** (248 mg, 2.30 mmol) as that just described gave (*S*)-**34** (300 mg, 71%). [α]_D_ −45 (*c* 1, CHCl_3_). ^1^H-NMR (270 MHz, CDCl_3_): *δ* = 7.69 (d, *J* = 7.8 Hz, 1H), 7.52 (s, 1H), 7.46 (t, *J* = 7.8 Hz, 1H), 7.23 (d, *J* = 7.8 Hz, 1H), 4.84 (q, *J* = 6.8 Hz, 1H), 3.78 (s, 3H), 1.66 (d, *J* = 6.8 Hz, 3H). ^13^C-NMR (67.5 MHz, CDCl_3_): *δ* = 180.1 (q, *^2^J_CF_* = 35.2 Hz), 171.9, 157.9, 131.2, 130.3, 123.5, 123.0, 118.7, 116.6 (q, *^1^J_CF_* = 291.1 Hz), 115.5, 72.7, 52.5, 18.4. HRMS (ESI): *m*/*z* calculated for C_12_H_11_F_3_O_4_ + H^+^ [M + H^+^]: 277.0688. Found: 277.0688.

**Methyl (*R*)-2-(3-(2,2,2-trifluoroacetyl)phenoxy)propanoate (*R*)-34.** The similar treatment of 2,2,2-trifluoro-1-(3-hydroxyphenyl)ethanone **32** (655 mg, 4.04 mmol) and methyl L-(−)-Lactate **5** (631 mg, 6.06 mmol) as that just described gave (*R*)-**34** (835 mg, 75%). [α]_D_ +45 (*c* 1, CHCl_3_). ^1^H-NMR (270 MHz, CDCl_3_): *δ* = 7.69 (d, *J* = 7.8 Hz, 1H), 7.53 (s, 1H), 7.46 (t, *J* = 7.8 Hz, 1H), 7.24 (d, *J* = 7.8 Hz, 1H), 4.85 (q, *J* = 6.8 Hz, 1H), 3.78 (s, 3H), 1.67 (d, *J* = 6.8 Hz, 3H). ^13^C-NMR (67.5 MHz, CDCl_3_): *δ* = 180.0 (q, *^2^J_CF_* = 35.2 Hz), 171.8, 157.9, 131.1, 130.3, 123.4, 122.9, 118.7, 116.5 (q, *^2^J_CF_* = 291.1 Hz), 115.4, 72.7, 52.4, 18.3. HRMS (ESI): *m*/*z* calculated for C_12_H_11_F_3_O_4_ + H^+^ [M + H^+^]: 277.0688. Found: 277.0685.

**(*S*)-2-(4-(2,2,2-Trifluoroacetyl)phenoxy)propanoic acid ((*S*)-35).** Methyl (*S*)-2-(4-(2,2,2-trifluoroacetyl)phenoxy)propanoate (*S*)-**33** (402 mg, 1.46 mmol) was dissolved in MeOH (18 mL) and H_2_O (2 mL), and then K_2_CO_3_ (605 mg, 4.37 mmol) was added. After the reaction mixture was stirred at reflux for 2 h, cooled to room temperature and then partitioned between ethyl acetate and water. The water layer was acidified by 1 M HCl and extracted by ethyl acetate. The organic layer was washed by H_2_O and brine, and dried over MgSO_4_, filtrated and concentrated to give (*S*)-**35** (400 mg, quant). [α]_D_ −31 (*c* 1, CHCl_3_). ^1^H-NMR (270 MHz, CDCl_3_): *δ* = 9.05 (1H, s), 7.97 (d, *J* = 8.9 Hz, 2H), 6.91 (d, *J* = 8.9 Hz, 2H), 4.85 (q, *J* = 6.8 Hz, 1H), 1.65 (d, *J* = 6.8 Hz, 3H). ^13^C-NMR (67.5 MHz, CDCl_3_): *δ* = 178.9 (q, *^2^J_CF_* = 35.2 Hz), 176.7, 162.9, 132.8, 123.7, 116.8 (q, *^1^J_CF_* = 291.6 Hz), 115.2, 72.0, 18.2. HRMS (ESI): *m*/*z* calculated for C_11_H_9_F_3_O_4_ + H^+^ [M + H^+^]: 263.0531. Found: 263.0543.

**(*R*)-2-(4-(2,2,2-Trifluoroacetyl)phenoxy)propanoic acid ((*R*)-35)**. The similar treatment of methyl (*R*)-2-(4-(2,2,2-trifluoroacetyl)phenoxy)propanoate (*R*)-**33** (476 mg, 1.72 mmol) as that just described gave (*R*)-**35** (428 mg, 95%). [α]_D_ +31 (*c* 1, CHCl_3_). ^1^H-NMR (270 MHz, CDCl_3_): *δ* = 7.99 (d, *J* = 8.9 Hz, 2H), 6.92 (d, *J* = 8.9 Hz, 2H), 4.86 (q, *J* = 6.8 Hz, 1H), 1.66 (d, *J* = 6.8 Hz, 3H). ^13^C-NMR (67.5 MHz, CDCl_3_): *δ* = 178.9 (q, *^2^J_CF_* = 34.6 Hz), 176.4, 162.9, 132.8, 123.8, 116.8 (q, *^1^J_CF_* = 291.3 Hz), 115.2, 71.9, 18.2. HRMS (ESI): *m*/*z* calculated for C_11_H_9_F_3_O_4_ + H^+^ [M + H^+^]: 263.0531. Found: 263.0541.

**(*S*)-2-(3-(2,2,2-Trifluoroacetyl)phenoxy)propanoic acid ((*S*)-36).** The similar treatment of methyl (*S*)-2-(3-(2,2,2-trifluoroacetyl)phenoxy)propanoate (*S*)-**34** (285 mg, 1.03 mmol) as that just described gave (*S*)-**36** (268 mg, 99%). [α]_D_ −28 (*c* 1, CHCl_3_). ^1^H-NMR (270 MHz, CDCl_3_): *δ* = 7.70 (d, *J* = 7.6 Hz, 1H), 7.56 (s, 1H), 7.47 (t, *J* = 8.1 Hz, 1H), 7.25 (d, *J* = 8.6 Hz, 1H), 4.88 (q, *J* = 6.8 Hz, 1H), 1.70 (d, *J* = 6.9 Hz, 3H). ^13^C-NMR (67.5 MHz, CDCl_3_): *δ* = 180.1 (q, *^2^J_CF_* = 35.2 Hz), 177.4, 157.7, 131.2, 130.4, 123.7, 122.8, 116.5 (q, *^1^J_CF_* = 291.3 Hz), 115.7, 72.2, 18.2. HRMS (ESI): *m*/*z* calculated for C_11_H_9_F_3_O_4_ + H^+^ [M + H^+^]: 263.0531. Found: 263.0543.

**(*R*)-2-(3-(2,2,2-Trifluoroacetyl)phenoxy)propanoic acid ((*R*)-36).** The similar treatment of methyl (*R*)-2-(3-(2,2,2-trifluoroacetyl)phenoxy)propanoate (*R*)-**34** (288 mg, 1.04 mmol) as that just described gave (*R*)-**36** (265 mg, 97%). [α]_D_ +28 (*c* 1, CHCl_3_). ^1^H-NMR (270 MHz, CDCl_3_): *δ* = 7.59 (d, *J* = 7.6 Hz, 1H), 7.46 (s, 1H), 7.36 (t, *J* = 8.1 Hz, 1H), 7.14 (d, *J* = 8.2 Hz, 1H), 4.78 (q, *J* = 6.8 Hz, 1H), 1.60 (d, *J* = 6.8 Hz, 3H). ^13^C-NMR (67.5 MHz, CDCl_3_): *δ* = 180.1 (q, *^2^J_CF_* = 35.2 Hz), 177.3, 157.7, 131.1, 130.4, 123.6, 122.8, 116.5 (q, *^1^J_CF_* = 291.6 Hz), 115.7, 72.2, 18.2. HRMS (ESI): *m*/*z* calculated for C_11_H_9_F_3_O_4_ + H^+^ [M + H^+^]: 263.0531. Found: 263.0538.

***tert*-Butyl (*S*)-2-(4-(2,2,2-trifluoroacetyl)phenoxy)propanoate ((*S*)-37).** To a solution of (*S*)-2-(4-(2,2,2-trifluoroacetyl)phenoxy)propanoic acid **35** (138 mg, 0.53 mmol), potassium carbonate (1.816 g, 13.1 mmol) and tetrabutylammonium bromide (169 mg, 0.53 mmol) in *N*,*N*-dimethylacetamide (2.6 mL), *tert*-butyl bromide (3.416 g, 24.9 mmol) was added dropwise at 0 °C. The reaction mixture was stirred at 55 °C for 2.5 h. After cooling to room temperature, the mixture was poured into cold water and extracted with ethyl acetate. The water layer was added 1 M HCl to make pH 1 and extracted with ethyl acetate, washed with brine, dried, filtrated and concentrated to recover (*S*)-**36** (21.8 mg, 16%). The organic layer was washed with H_2_O, brine, dried over MgSO4, filtrated and evaporated. The residue was purified by column chromatography (ethyl acetate/*n*-hexane, 1:6) to give (*S*)-**37** (111 mg, 66%). [α]_D_ −38 (*c* 1, CHCl_3_). ^1^H-NMR (270 MHz, CDCl_3_): *δ* = 8.04 (d, *J* = 8.9 Hz, 2H), 6.96 (d, *J* = 8.9 Hz, 2H), 4.75 (q, *J* = 6.8 Hz, 1H), 1.64 (d, *J* = 6.8 Hz, 3H), 1.45 (s, 9H). ^13^C-NMR (67.5 MHz, CDCl_3_): *δ* = 178.9 (q, *^2^J_CF_* = 35.2 Hz), 170.1, 163.5, 132.7, 123.3, 116.9 (q, *^1^J_CF_* = 291.1 Hz), 115.2, 82.7, 73.0, 27.9, 18.2. HRMS (ESI): *m*/*z* calculated for C_15_H_17_F_3_O_4_ + H^+^ [M + H^+^]: 319.1157. Found: 319.1158.

***tert*-Butyl (*R*)-2-(4-(2,2,2-trifluoroacetyl)phenoxy)propanoate ((*R*)-37).** The similar treatment of (*R*)-2-(4-(2,2,2-trifluoroacetyl)phenoxy)propanoic acid (*R*)-**35** (428 mg, 1.63 mmol) as that just described gave (*R*)-**37** (363 mg, 70%). [α]_D_ +38 (*c* 1, CHCl_3_). ^1^H-NMR (270 MHz, CDCl_3_): *δ* = 7.96 (d, *J* = 8.9 Hz, 2H), 6.88 (d, *J* = 8.9 Hz, 2H), 4.67 (q, *J* = 6.8 Hz, 1H), 1.55 (d, *J* = 6.8 Hz, 3H), 1.36 (s, 9H). ^13^C-NMR (67.5 MHz, CDCl_3_): *δ* = 178.8 (q, *^2^J_CF_* = 34.6 Hz), 170.1, 163.5, 132.6, 123.2, 116.8 (q, *^1^J_CF_* = 291.1 Hz), 115.2, 82.6, 73.0, 27.8, 18.2. HRMS (ESI): *m*/*z* calculated for C_15_H_17_F_3_O_4_ + H^+^ [M + H^+^]: 319.1157. Found: 319.1147.

***tert*-Butyl (*S*)-2-(3-(2,2,2-trifluoroacetyl)phenoxy)propanoate ((*S*)-38).** The similar treatment of (*S*)-2-(3-(2,2,2-trifluoroacetyl)phenoxy)propanoic acid (*S*)-**36** (310 mg, 1.18 mmol) as that just described gave (*S*)-**36** (149 mg, 48%) and (*S*)-**38** (141 mg, 38%). [α]_D_ −53 (*c* 1, CHCl_3_). ^1^H-NMR (270 MHz, CDCl_3_): *δ* = 7.67 (d, *J* = 7.8 Hz, 1H), 7.51 (s, 1H), 7.45 (t, *J* = 7.8 Hz, 1H), 7.25 (d, *J* = 7.8 Hz, 1H), 4.69 (q, *J* = 6.8 Hz, 1H), 1.62 (d, *J* = 6.8 Hz, 3H), 1.45 (s, 9H). ^13^C-NMR (67.5 MHz, CDCl_3_): *δ* = 180.2 (q, *^2^J_CF_* = 34.6 Hz), 170.6, 158.2, 131.0, 130.2, 123.3, 123.2, 116.6 (q, *^1^J_CF_* = 291.1 Hz), 115.0, 82.5, 73.0, 27.8, 18.3. HRMS (ESI): *m*/*z* calculated for C_15_H_17_F_3_O_4_ + H^+^ [M + H^+^]: 319.1157. Found: 319.1159.

***tert*-Butyl (*R*)-2-(3-(2,2,2-trifluoroacetyl)phenoxy)propanoate((*R*)-38).** The similar treatment of (*R*)-2-(3-(2,2,2-trifluoroacetyl)phenoxy)propanoic acid (*R*)-**36** (344 mg, 1.31 mmol) as that just described gave (*R*)-**36** (167 mg, 48%) and (*R*)-**38** (153 mg, 37%). [α]_D_ +53 (*c* 1, CHCl_3_). ^1^H-NMR (270 MHz, CDCl_3_): *δ* = 7.68 (d, *J* = 7.8 Hz, 1H), 7.51 (s, 1H), 7.45 (t, *J* = 7.8 Hz, 1H), 7.24 (d, *J* = 7.8 Hz, 1H), 4.69 (q, *J* = 6.8 Hz, 1H), 1.62 (d, *J* = 6.8 Hz, 3H), 1.45 (s, 9H). ^13^C-NMR (67.5 MHz, CDCl_3_): *δ* = 180.1 (q, *^2^J_CF_* = 35.2 Hz), 170.6, 158.2, 131.0, 130.2, 123.3, 123.2, 116.6 (q, *^1^J_CF_* = 291.1 Hz), 115.0, 82.5, 73.0, 27.8, 18.3. HRMS (ESI): *m*/*z* calculated for C_15_H_17_F_3_O_4_ + H^+^ [M + H^+^]: 319.1157. Found: 319.1183.

**2-(1,3-Dithian-2-yl)phenol 40.** Salicylaldehyde **39** (0.62 g, 5.06 mmol) and iodine (130 mg, 0.51 mmol) were dissolved in CH_2_Cl_2_ (25 mL) and then 1,3-propanedithiol (0.65 g, 6.00 mmol) was added. After the reaction mixture was stirred at room temperature for 1 h, then quenched aqueous sodium thiosulfate (0.5 M, 10 mL). The mixture was extracted with CH_2_Cl_2_, washed with brine, dried over MgSO_4_, filtrated and concentrated to give 2-(1,3-dithian-2-yl)phenol **40** (1.02 g, 95%). ^1^H-NMR (270 MHz, CDCl_3_): *δ* = 7.29 (dd, *J* = 7.9, 1.6 Hz, 1H), 7.21 (td, *J* = 7.7, 1.6 Hz, 1H), 6.88 (dq, *J* = 7.4, 2.0 Hz, 2H), 6.33 (s, 1H), 5.40 (s, 1H), 3.07 (td, *J* = 13.6, 3.0 Hz, 2H), 2.91 (dt, *J* = 14.2, 3.6 Hz, 2H), 2.26–2.13 (m, 1H), 2.00–1.87 (m, 1H). ^13^C-NMR (67.5 MHz, CDCl_3_): *δ* = 154.5, 130.1, 129.1, 123.5, 120.8, 117.3, 47.4, 31.6, 24.8. HRMS (ESI): *m*/*z* calculated for C_10_H_12_O_2_S_2_ + H^+^ [M + H^+^]: 213.0408. Found: 213.0383.

**Methyl (*S*)-2-(2-(1,3-dithian-2-yl)phenoxy)propanoate ((*S*)-41).** 2-(1,3-Dithian-2-yl)phenol **40** (1.27 g) phenol, methyl D-(+)-lactate **4** (0.94 g, 8.99 mmol) and PPh_3_ (1.90 g, 7.22 mmol) were dissolved in dry CH_2_Cl_2_ (30 mL). After stirring for 10 min at 0 °C, DEAD (1.57 g, 9.02 mmol) was slowly added. The reaction mixture was stirred overnight at room temperature, and then partitioned between water and CH_2_Cl_2_. The organic layer was washed with brine, dried over MgSO_4_, filtered and concentrated. The residue was purified by column chromatography (ethyl acetate/*n*-hexane, 1:9) to give (*S*)-**41** (1.48 g, 98%). [α]_D_ +31 (*c* 1, CHCl_3_). ^1^H-NMR (270 MHz, CDCl_3_): *δ* = 7.60 (d, *J* = 7.6 Hz, 1H), 7.19 (t, *J* = 7.6 Hz, 1H), 7.00 (t, *J* = 7.6 Hz, 1H), 6.75 (d, *J* = 7.6 Hz, 1H), 5.76 (s, 1H), 4.78 (q, *J* = 6.8 Hz, 1H), 3.75 (s, 3H), 3.11 (m, 2H), 2.88 (m, 2H), 2.16 (m, 1H), 1.95 (m, 1H), 1.67 (d, *J* = 6.8 Hz, 3H). ^13^C-NMR (67.5 MHz, CDCl_3_): *δ* = 172.3, 153.8, 129.4, 129.2, 128.6, 122.2, 113.4, 73.9, 52.2, 43.8, 32.3, 32.2, 25.3, 18.5. HRMS (ESI): *m*/*z* calculated for C_14_H_18_O_3_S_2_ + Na^+^ [M + Na^+^]: 321.0595. Found: 321.0597.

**Methyl (*R*)-2-(2-(1,3-dithian-2-yl)phenoxy)propanoate ((*R*)-41).** 2-(1,3-Dithian-2-yl)phenol **40** (1.27 g) as that just described gave (*R*)-**41** (1.47 g, 98%). [α]_D_ −31 (*c* 1, CHCl_3_). ^1^H-NMR (270 MHz, CDCl_3_): *δ* = 7.60 (d, *J* = 7.6 Hz, 1H), 7.19 (t, *J* = 7.6 Hz, 1H), 7.00 (t, *J* = 7.6 Hz, 1H), 6.75 (d, *J* = 7.6 Hz, 1H), 5.76 (s, 1H), 4.78 (q, *J* = 6.8 Hz, 1H), 3.75 (s, 3H), 3.11 (m, 2H), 2.90 (m, 2H), 2.16 (m, 1H), 1.95 (m, 1H), 1.67 (d, *J* = 6.9 Hz, 3H). ^13^C-NMR (67.5 MHz, CDCl_3_): *δ* = 172.3, 153.8, 129.4, 129.2, 128.7, 122.3, 113.4, 74.0, 52.2, 43.8, 32.3, 32.2, 25.3, 18.6. HRMS (ESI): *m*/*z* calculated for C_14_H_18_O_3_S_2_ + Na^+^ [M + Na^+^]: 321.0595. Found: 321.0598.

**(*S*)-2-(2-(1,3-Dithian-2-yl)phenoxy)propanoic acid (*S*)-42.** Methyl (*S*)-2-(2-(1,3-dithian-2-yl)phenoxy)propanoate (*S*)-**41** (1.51 g, 5.06 mmol) was dissolved in MeOH (30 mL) and H_2_O (3.3 mL), and then K_2_CO_3_ (700 mg, 5.06 mmol) was added. After the reaction mixture was stirred at reflux for 2 h, cooled to room temperature and then partitioned between ethyl acetate and water. The water layer was acidified by 1 M HCl aq and extracted by ethyl acetate. The organic layer was washed by H_2_O and brine, and dried over MgSO_4_, filtrated and concentrated to give (*S*)-**42** (1.63 g, quant). [α]_D_ +9 (*c* 1, CHCl_3_). ^1^H-NMR (270 MHz, CDCl_3_): *δ* = 7.55 (d, *J* = 7.6 Hz, 1H), 7.26 (t, *J* = 7.6 Hz, 1H), 7.03 (t, *J* = 7.6 Hz, 1H), 6.82 (d, *J* = 7.6 Hz, 1H), 5.62 (s, 1H), 4.88 (q, *J* = 6.8 Hz, 1H), 3.12 (m, 2H), 2.92 (m, 2H), 2.19 (m, 1H), 1.96 (m, 1H), 1.71 (d, *J* = 6.8 Hz, 3H). ^13^C-NMR (67.5 MHz, CDCl_3_): *δ* = 174.9, 153.3, 129.6, 129.5, 128.1, 122.6, 112.8, 73.0, 45.4, 32.2, 32.1, 25.3, 18.0. HRMS (ESI): *m*/*z* calculated for C_13_H_16_O_3_S_2_ + H^+^ [M + H^+^]: 285.0619. Found: 285.0591.

**(*R*)-2-(2-(1,3-dithian-2-yl)phenoxy)propanoic acid (*R*)-42.** The similar treatment of methyl (*R*)-2-(2-(1,3-dithian-2-yl)phenoxy)propanoate (*R*)-**41** (1.56 g, 5.22 mmol) as that just described gave (*S*)-**42** (1.69 g, quant). [α]_D_ −9 (*c* 1, CHCl_3_). ^1^H-NMR (270 MHz, CDCl_3_): *δ* = 7.56 (d, *J* = 7.6 Hz, 1H), 7.24 (t, *J* = 7.6 Hz, 1H), 7.02 (t, *J* = 7.6 Hz, 1H), 6.81 (d, *J* = 7.6 Hz, 1H), 5.65 (s, 1H), 4.85 (q, *J* = 6.8 Hz, 1H), 3.11 (m, 2H), 2.90 (m, 2H), 2.18 (m, 1H), 1.95 (m, 1H), 1.71 (d, *J* = 6.8 Hz, 3H). ^13^C-NMR (67.5 MHz, CDCl_3_): *δ* = 175.8, 153.3, 129.5, 129.5, 128.2, 122.5, 112.9, 73.1, 45.0, 32.2, 32.1, 25.2, 18.1. HRMS (ESI): *m*/*z* calculated for C_13_H_16_O_3_S_2_ + H^+^ [M + H^+^]: 285.0619. Found: 285.0608.

***tert*-Butyl (*S*)-2-(2-(1,3-dithian-2-yl)phenoxy)propanoate (*S*)-43.** To a solution of (*S*)-2-(2-(1,3-dithian-2-yl)phenoxy)propanoic acid (1.63 g, 5.72 mmol) in *N*,*N*-dimethylacetamide (29 mL) in the presence of potassium carbonate (19.8 g, 143 mmol) and tetrabutylammonium bromide (1.85 g, 5.73 mmol) at 0 °C, *tert*-butyl bromide (37.6 g, 274 mmol) was added dropwise and the reaction mixture was stirred at 55 °C for 2.5 h. After cooling to room temperature, the mixture was added into cold water and extracted with ethyl acetate. The organic layer was washed with H_2_O, brine, dried over MgSO_4_, filtrated and evaporated. The residue was purified by column chromatography (ethyl acetate/*n*-hexane, 1:6) to give (*S*)-**43** (1.61 g, 83%). [α]_D_ +25 (*c* 1, CHCl_3_). ^1^H-NMR (270 MHz, CDCl_3_): *δ* = 7.59 (d, *J* = 7.8 Hz, 1H), 7.18 (t, *J* = 7.8 Hz, 1H), 6.97 (t, *J* = 7.8 Hz, 1H), 6.74 (d, *J* = 7.8 Hz, 1H), 5.75 (s, 1H), 4.64 (q, *J* = 6.8 Hz, 1H), 3.11 (m, 2H), 2.88 (m, 2H), 2.16 (m, 1H), 1.93 (m, 1H), 1.63 (d, *J* = 6.8Hz, 3H), 1.43 (s, 9H). ^13^C-NMR (67.5 MHz, CDCl_3_): *δ* = 171.0, 153.9, 129.3, 129.0, 128.3, 121.8, 112.8, 81.8, 73.9, 43.8, 32.3, 32.2, 27.9, 25.4, 18.4. HRMS (ESI): *m*/*z* calculated for C_17_H_24_O_3_S_2_ + H^+^ [M + H^+^]: 341.1245. Found: 341.1237.

***tert*-Butyl (*R*)-2-(2-(1,3-dithian-2-yl)phenoxy)propanoate (*R*)-43.** The similar treatment of (*R*)-2-(2-(1,3-dithian-2-yl)phenoxy)propanoic acid **42** (1.69 g, 5.93 mmol) as that just described gave (*R*)-**43** (1.65 g, 82%). [α]_D_ −25 (*c* 1, CHCl_3_). ^1^H-NMR (270 MHz, CDCl_3_): *δ* = 7.59 (d, *J* = 7.8 Hz, 1H), 7.18 (t, *J* = 7.8 Hz, 1H), 6.97 (t, *J* = 7.8 Hz, 1H), 6.73 (d, *J* = 7.8 Hz, 1H), 5.75 (s, 1H), 4.64 (q, *J* = 6.8 Hz, 1H), 3.11 (m, 2H), 2.88 (m, 2H), 2.16 (m, 1H), 1.95 (m, 1H), 1.63 (d, *J* = 6.8 Hz, 3H), 1.43 (s, 9H). ^13^C-NMR (67.5 MHz, CDCl_3_): *δ* = 171.0, 153.9, 129.3, 129.0, 128.3, 121.8, 112.8, 81.8, 73.9, 43.8, 32.4, 32.2, 27.9, 25.4, 18.4. HRMS (ESI): *m*/*z* calculated for C_17_H_24_O_3_S_2_ + H^+^ [M + H^+^]: 341.1245. Found: 341.1239.

***tert*-Butyl (S)-2-(2-formylphenoxy)propanoate (*S*)-44.***tert*-Butyl (*S*)-2-(2-(1,3-dithian-2-yl)phenoxy)propanoate (*S*)-**43** (94.3 mg, 0.28 mmol) and sodium hydrogen carbonate (465 mg, 5.54 mmol) were dissolved in acetonitrile (10 mL) and water (2 mL), and then iodomethane (392 mg, 2.8 mmol)was added. After the reaction mixture was stirred at room temperature for 24 h, iodomethane (196 mg, 1.4 mmol) was added. After stirred at room temperature for 24 h, the reaction mixture extracted with ethyl acetate. The organic layer was washed with brine, dried over MgSO4, filtrated and concentrated. The residue was purified by column chromatography (ethyl acetate/*n*-hexane, 1:6) to give (*S*)-**44** (52.3 mg, 78%). [α]_D_ +14 (*c* 1, CHCl_3_). ^1^H-NMR (270 MHz, CDCl_3_): *δ* = 10.57 (s, 1H), 7.85 (d, *J* = 7.9 Hz, 1H), 7.49 (t, *J* = 7.9 Hz, 1H), 7.04 (t, *J* = 7.9 Hz, 1H), 6.85 (d, *J* = 7.9 Hz, 1H), 4.77 (q, *J* = 6.8 Hz, 1H), 1.66 (d, *J* = 6.8 Hz, 3H), 1.42 (s, 9H). ^13^C-NMR (67.5 MHz, CDCl_3_): *δ* = 189.7, 170.4, 160.2, 135.5, 128.2, 125.4, 121.4, 113.2, 82.3, 73.5, 27.8, 18.2. HRMS (ESI): *m*/*z* calculated for C_14_H_18_O_4_ + H^+^ [M + H^+^]: 251.1283. Found: 251.1312.

***tert*-Butyl (*R*)-2-(2-formylphenoxy)propanoate (*R*)-44.** The similar treatment of *tert*-butyl (*R*)-2-(2-(1,3-dithian-2-yl)phenoxy)propanoate (*R*)-**43** (156 mg, 0.46 mmol) as that just described gave (*R*)-**44** (90.4 mg, 79%). [α]_D_ −14 (*c* 1, CHCl_3_). ^1^H-NMR (270 MHz, CDCl_3_): *δ* = 10.58 (s, 1H), 7.84 (d, *J* = 7.6 Hz, 1H), 7.50 (t, *J* = 7.6 Hz, 1H), 7.04 (t, *J* = 7.6 Hz, 1H), 6.85 (d, *J* = 7.6 Hz, 1H), 4.78 (q, *J* = 6.8 Hz, 1H), 1.66 (d, *J* = 6.8 Hz, 3H), 1.42 (s, 9H). ^13^C-NMR (67.5 MHz, CDCl_3_): *δ* = 189.7, 170.4, 160.2, 135.5, 128.2, 125.4, 121.4, 113.2, 82.3, 73.5, 27.8, 18.2. HRMS (ESI): *m*/*z* calculated for C_14_H_18_O_4_ + H^+^ [M + H^+^]: 251.1283. Found: 251.1292.

***tert*-Butyl (*S*)-2-(2-(2,2,2-trifluoroacetyl)phenoxy)propanoate (*S*)-45.** To the solution of tert-butyl (*S*)-2-(2-formylphenoxy)propanoate (*S*)-**44** (591 mg, 2.04 mmol) in dimethylacetamide (7.9 mL), (trifluoromethyl)trimetylsilane (579 mg, 4.07 mmol) and potassium carbonate (16.3 mg, 0.118 mmol) were added. The reaction mixture was and stirred at room temperature for 4 h and treated with 1 M HCl (1 mL) at room temperature for 4 h, then extracted with ethyl acetate. The organic layer was washed with brine twice, dried over MgSO4, filtrated and concentrated to afford crude material. To crude phenylethanol derivative (784 mg) was dissolved in CH_2_Cl_2_ (13 mL), Dess-Martin periodinane (1.096 g, 2.58 mmol) was added at room temperature. The reaction mixture was stirred at room temperature for 3 h, then washed with water and brine. The organic layer was dried over MgSO_4_, filtrated and concentrated. The residue was subjected to column chromatography (hexane/AcOEt, 8:1) to give (*S*)-**45** (636 mg, 85%). [α]_D_ +25 (*c* 1, CHCl_3_). ^1^H-NMR (270 MHz, CDCl_3_): *δ* = 7.54 (d, *J* = 7.6 Hz, 1H), 7.41 (t, *J* = 7.6 Hz, 1H), 6.95 (t, *J* = 7.6 Hz, 1H), 6.71 (d, *J* = 7.6 Hz, 1H), 4.61 (q, *J* = 6.8 Hz, 1H), 1.51 (d, *J* = 6.8 Hz, 3H), 1.30 (s, 9H). ^13^C-NMR (67.5 MHz, CDCl_3_): *δ* = 183.6 (q, *^2^J_CF_* = 37.4 Hz), 170.2, 157.5, 135.3, 131.5, 122.6, 121.2, 116.1 (q, *^1^J_CF_* = 290.5 Hz), 112.8, 82.4, 73.7, 27.8, 17.8. HRMS (ESI): *m*/*z* calculated for C_15_H_17_F_3_O_4_ + H^+^ [M + H^+^]: 319.1157. Found: 319.1171.

***tert*-Butyl (*R*)-2-(2-(2,2,2-trifluoroacetyl)phenoxy)propanoate (*R*)-45.** The similar treatment of *tert*-butyl (*R*)-2-(2-formylphenoxy)propanoate (*R*)-**44** (577 mg, 2.30 mmol) as that just described gave (*R*)-**45** (651 mg, 89%). [α]_D_ −25 (*c* 1, CHCl_3_). ^1^H-NMR (270 MHz, CDCl_3_): *δ* = 7.65 (d, *J* = 7.6 Hz, 1H), 7.53 (t, *J* = 7.6 Hz, 1H), 7.06 (t, *J* = 7.6 Hz, 1H), 6.83 (d, *J* = 7.6 Hz, 1H), 4.72 (q, *J* = 6.8 Hz, 1H), 1.62 (d, *J* = 6.8 Hz, 3H), 1.41 (s, 9H). ^13^C-NMR (67.5 MHz, CDCl_3_): *δ* = 183.6 (q, *^2^J_CF_* = 37.4 Hz), 170.2, 157.5, 135.3, 131.6, 122.6, 121.2, 116.1 (q, *^1^J_CF_* = 290.5 Hz), 112.8, 82.4, 73.7, 27.8, 17.8. HRMS (ESI): *m*/*z* calculated for C_15_H_17_F_3_O_4_ + H^+^ [M + H^+^]: 319.1157. Found: 319.1141.

***tert*-Butyl (*S*)-2-(4-(2,2,2-trifluoro-1-(hydroxyimino)ethyl)phenoxy)propanoate ((*S*)-46).** A solution of *tert*-butyl (*S*)-2-(4-(2,2,2-trifluoroacetyl)phenoxy)propanoate (*S*)-**37** (252 mg, 0.79 mmol) and hydroxylamine hydrochloride (60.7 mg, 0.87 mmol) in ethanol (0.44 mL) and dry pyridine (0.79 mL) was heated at 60 °C for 8 h. The reaction mixture was partitioned between water and ether, and the organic layer was washed with 1 M HCl, saturated NaHCO_3_ and brine. The organic layer was dried over MgSO_4_, filtrated and concentrated. The residue was purified by column chromatography (ethyl acetate/*n*-hexane, 1:6) to give (*S*)-**46** (275 mg, quant). The product was mixture of *syn*- and *anti*- isomers. [α]_D_ −38 (*c* 1, CHCl_3_). ^1^H-NMR (270 MHz, CDCl_3_): *δ* = 8.89 and 8.75 (s, 1H), 7.44 and 7.34 (d × 2, *J* = 8.9 Hz, 2H), 6.86 and 6.81 (d × 2, *J* = 8.9 Hz, 2H), 4.60 and 4.59 (q × 2, *J* = 6.8 Hz, 1H), 1.53 (d, *J* = 6.8 Hz, 3H), 1.37 (s, 9H). ^13^C-NMR (67.5 MHz, CDCl_3_): *δ* = 171.0, 159.3, 146.9 (q, *^2^J_CF_* = 32.4 Hz), 130.5, 120.8 (q, *^1^J_CF_* = 274.9 Hz), 118.6, 114.9, 82.4, 72.9, 27.9, 18.3. HRMS (ESI): *m*/*z* calculated for C_15_H_18_F_3_NO_4_ + H^+^ [M + H^+^]: 334.1266. Found: 334.1256.

***tert*-Butyl (*R*)-2-(4-(2,2,2-trifluoro-1-(hydroxyimino)ethyl)phenoxy)propanoate ((*R*)-46).** The similar treatment of *tert*-butyl (*R*)-2-(4-(2,2,2-trifluoroacetyl)phenoxy)propanoate (*R*)-**37** (459 mg, 1.44 mmol) as that just described gave (*R*)-**46** (490 mg, quant). [α]_D_ +38 (*c* 1, CHCl_3_). ^1^H-NMR (270 MHz, CDCl_3_): *δ* = 8.36 and 8.19 (s, 1H), 7.50 and 7.42 (d × 2, *J* = 7.8 Hz, 2H), 6.93 and 6.88 (d × 2, *J* = 8.9 Hz, 2H), 4.67 and 4.66 (q × 2, *J* = 6.8 Hz, 1H), 1.60 (d, *J* = 6.8 Hz, 3H), 1.44 (s, 9H). ^13^C-NMR (67.5 MHz, CDCl_3_): *δ* = 171.0, 159.3, 146.9 (q, *^2^J_CF_* = 32.4 Hz), 130.5, 120.8 (q, *^1^J_CF_* = 274.9 Hz), 118.6, 114.9, 82.4, 72.9, 27.9, 18.3. HRMS (ESI): *m*/*z* calculated for C_15_H_18_F_3_NO_4_ + H^+^ [M + H^+^]: 334.1266. Found: 334.1236.

***tert*-Butyl (*S*)-2-(3-(2,2,2-trifluoro-1-(hydroxyimino)ethyl)phenoxy)propanoate ((*S*)-47).** The similar treatment of *tert*-butyl (*S*)-2-(3-(2,2,2-trifluoroacetyl)phenoxy)propanoate (*S*)-**38** (81.7 mg, 0.26 mmol) as that just described gave (*S*)-**47** (85.7 mg, quant). [α]_D_ −40 (*c* 1, CHCl_3_). ^1^H-NMR (270 MHz, CDCl_3_): *δ* = 7.36 and 7.30 t × 2, *J* = 7.8 Hz, 1H), 7.09 (d, *J* = 7.8 Hz, 1H), 6.97 (m, 2H), 4.66 (q, *J* = 6.8 Hz, 1H), 1.60 and 1.59 (d × 2, *J* = 6.8 Hz, 3H), 1.43 and 1.42 (s × 2, 9H). ^13^C-NMR (67.5 MHz, CDCl_3_): *δ* = 171.3, 157.6, 147.3 (q, *^2^J_CF_* = 32.4 Hz), 129.7 and 129.6, 127.2, 121.5, 120.6 (q, *^1^J_CF_* = 274.3 Hz), 117.4 and 117.0, 115.4 and 115.2, 82.5, 73.1, 27.8, 18.4. HRMS (ESI): *m*/*z* calculated for C_15_H_18_F_3_NO_4_ + H^+^ [M + H^+^]: 334.1266. Found: 334.1265.

***tert*-Butyl (*R*)-2-(3-(2,2,2-trifluoro-1-(hydroxyimino)ethyl)phenoxy)propanoate ((*R*)-47).** The similar treatment of *tert*-butyl (*R*)-2-(3-(2,2,2-trifluoroacetyl)phenoxy)propanoate (*R*)-**38** (97.2 mg, 0.31 mmol) as that just described gave (*R*)-**47** (86.6 mg, 85%). [α]_D_ +40 (*c* 1, CHCl_3_). ^1^H-NMR (270 MHz, CDCl_3_): *δ* = 7.37 and 7.30 (t × 2, *J* = 7.8 Hz, 2H), 7.08 (d, *J* = 7.8 Hz, 1H), 6.97 (m, 2H), 4.65 (q, *J* = 6.8 Hz, 1H), 1.60 (d, *J* = 6.8 Hz, 3H), 1.43 and 1.42 (s × 2, 9H). ^13^C-NMR (67.5 MHz, CDCl_3_): *δ* = 171.1, 157.6, 147.6 (q, *^2^J_CF_* = 32.4 Hz), 129.7 and 129.5, 127.1, 121.4, 120.5 (q, *^1^J_CF_* = 274.9 Hz), 117.4 and 117.1, 115.3 and 115.1, 82.3, 73.1, 27.8, 18.4. HRMS (ESI): *m*/*z* calculated for C_15_H_18_F_3_NO_4_ + H^+^ [M + H^+^]: 334.1266. Found: 334.1257.

***tert*-Butyl (*S*)-2-(2-(2,2,2-trifluoro-1-(hydroxyimino)ethyl)phenoxy)propanoate ((*S*)-48).** The similar treatment of *tert*-butyl (*S*)-2-(2-(2,2,2-trifluoroacetyl)phenoxy)propanoate (*S*)-**45** (309 mg, 0.97 mmol) as that just described gave (*S*)-**48** (331 mg, quant). [α]_D_ +8 (*c* 1, CHCl_3_). ^1^H-NMR (270 MHz, CDCl_3_): *δ* = 7.37 and 7.36 (t × 2, *J* = 7.6 Hz, 1H), 7.27 and 7.21 (d × 2, *J* = 7.6 Hz, 1H), 7.03 and 6.99 (t × 2, *J* = 7.6 Hz, 1H), 6.80 and 6.75 (d × 2, *J* = 7.6 Hz, 1H), 4.62 (q, *J* = 6.8 Hz, 1H), 1.57 and 1.54 (d × 2, *J* = 6.8 Hz, 3H), 1.41 (s, 9H). ^13^C-NMR (67.5 MHz, CDCl_3_): *δ* = 171.2 and 171.0, 156.5, 155.1, 147.6 and 146.7 (q × 2, *^2^J_CF_* = 33.5 Hz), 131.6, 131.0, 129.8, 121.0, 117.9 (q, *^1^J_CF_* = 287.2 Hz), 116.3, 112.2 and 111.7, 82.3 and 82.2, 73.5 and 73.4, 27.8, 18.2 and 18.0. HRMS (ESI): *m/z* calculated for C_15_H_18_F_3_NO_4_ + H^+^ [M + H^+^]: 334.1266. Found: 334.1246.

***tert*-Butyl (*R*)-2-(2-(2,2,2-trifluoro-1-(hydroxyimino)ethyl)phenoxy)propanoate ((*R*)-48).** The similar treatment of *tert*-butyl (*R*)-2-(2-(2,2,2-trifluoroacetyl)phenoxy)propanoate (*R*)-**45** (291 mg, 0.92 mmol) as that just described gave (*R*)-**48** (299 mg, 98%). [α]_D_ −8 (*c* 1, CHCl_3_). ^1^H-NMR (270 MHz, CDCl_3_): *δ* = 7.37 (m, 1H), 7.27 and 7.21 (d, *J* = 7.6 Hz, 1H), 7.03 and 6.98 (t, *J* = 7.6 Hz, 1H), 6.80 and 6.74 (d, *J* = 7.6 Hz, 1H), 4.62 (q, *J* = 6.8 Hz, 1H), 1.57 and 1.54 (d, *J* = 6.8 Hz, 3H), 1.41 (s, 9H). ^13^C-NMR (67.5 MHz, CDCl_3_): *δ* = 171.2 and 171.1, 156.5, 155.1, 147.5 and 146.5 (q × 2, *^2^J_CF_* = 34.1 Hz), 131.6, 131.0, 129.8, 121.0, 120.5 and 118.2 (q × 2, *^1^J_CF_* = 287.2 Hz), 116.3, 112.2 and 111.7, 82.3 and 82.2, 73.5 and 73.4, 27.8, 18.1 and 18.0. HRMS (ESI): *m*/*z* calculated for C_15_H_18_F_3_NO_4_ + H^+^ [M + H^+^]: 334.1266. Found: 334.1261.

***tert*-Butyl (*S*)-2-(4-(2,2,2-trifluoro-1-((tosyloxy)imino)ethyl)phenoxy)propanoate ((*S*)-49).***tert*-Butyl (*S*)-2-(4-(2,2,2-trifluoro-1-(hydroxyimino)ethyl)phenoxy)propanoate (*S*)-**46** (268 mg, 0.80 mmol), triethylamine (203 mg, 2.01 mmol) and DMAP (4.9 mg, 0.040 mmol) were suspended in CH_2_Cl_2_ (1.5 mL) at 0 °C. *p*-Toluenesulfonyl chloride (168 mg, 0.88 mmol) was added at 0 °C. The reaction mixture was stirred at room temperature for 45 min, then washed with water. The organic layer was dried over MgSO_4_, filtrated and concentrated to give (*S*)-**49** (418 mg, quant). The product was mixture of *syn*- and *anti*- isomers. [α]_D_ −20 (*c* 1, CHCl_3_). ^1^H-NMR (270 MHz, CDCl_3_): *δ* = 7.88 (d, *J* = 8.2 Hz, 2H), 7.45–7.35 (m, 4H), 6.92 and 6.87 (d, *J* = 8.2 Hz, 2H), 4.68 (q, *J* = 6.8 Hz, 1H), 2.46 and 2.45 (s × 2, 3H), 1.61 and 1.60 (d × 2, *J* = 6.8 Hz, 3H), 1.44 (s, 9H). ^13^C-NMR (67.5 MHz, CDCl_3_): *δ* = 170.5 and 170.4, 160.6 and 160.2, 152.9 (q, *^2^J_CF_* = 27.9 Hz), 146.0 and 145.9, 131.4 and 131.1, 130.5, 129.8, 129.1, 129.0, 128.9, 120.3, 119.7 and 117.4 (q, *^1^J_CF_* = 276.0 Hz), 116.9, 115.0, 82.3, 72.8 and 72.7, 27.8 and 27.7, 21.6, 18.2. HRMS (ESI): *m*/*z* calculated for C_22_H_24_F_3_NO_6_S + H^+^ [M + H^+^]: 488.1355. Found: 488.1372.

***tert*-Butyl (*R*)-2-(4-(2,2,2-trifluoro-1-((tosyloxy)imino)ethyl)phenoxy)propanoate ((*R*)-49).** The similar treatment of tert-butyl (*R*)-2-(4-(2,2,2-trifluoro-1-(hydroxyimino)ethyl)phenoxy)-propanoate (*R*)-**46** (331 mg, 0.99 mmol) as that just described gave (*R*)-**49** (473 mg, 98%). [α]_D_ +20 (*c* 1, CHCl_3_). ^1^H-NMR (270 MHz, CDCl_3_): *δ* = 7.89 (d, *J* = 7.8 Hz, 2H), 7.38 (m, 4H), 6.87 (d, *J* = 7.8 Hz, 2H), 4.65 (q, *J* = 6.8 Hz, 1H), 2.46 (s, 3H), 1.60 (d, *J* = 6.8 Hz, 3H), 1.44 (s, 9H). ^13^C-NMR (67.5 MHz, CDCl_3_): *δ* = 170.5 and 170.4, 160.6 and 160.2, 153.2 (q, *^2^J_CF_* = 29.9 Hz), 146.0 and 145.9, 131.4 and 131.1, 130.5, 129.8, 129.1, 128.9, 120.3, 118.6 (q, *^1^J_CF_* = 283.8 Hz), 116.9, 115.0, 82.3, 72.8 and 72.7, 27.7 and 27.8, 21.6, 18.2. HRMS (ESI): *m*/*z* calculated for C_22_H_24_F_3_NO_6_S + H^+^ [M + H^+^]: 488.1355. Found: 488.1380.

***tert*-Butyl (*S*)-2-(3-(2,2,2-trifluoro-1-((tosyloxy)imino)ethyl)phenoxy)propanoate ((*S*)-50)**. The similar treatment of *tert*-butyl (*S*)-2-(3-(2,2,2-trifluoro-1-(hydroxyimino)ethyl)phenoxy)-propanoate (*S*)-**47** (277 mg, 0.83 mmol) as that just described gave (*S*)-**50** (431 mg, quant). [α]_D_ −34 (*c* 1, CHCl_3_). ^1^H-NMR (270 MHz, CDCl_3_): *δ* = 7.88 (d, *J* = 8.6 Hz, 2H), 7.35 (m, 3H), 7.00 (d, *J* = 7.8 Hz, 2H), 6.97 (d, *J* = 7.8 Hz, 2H), 6.85 (s, 1H), 4.62 (q, *J* = 6.8 Hz, 1H), 2.48 (s, 3H), 1.60 (d, *J* = 6.8 Hz, 3H), 1.42 (s, 9H). ^13^C-NMR (67.5 MHz, CDCl_3_): *δ* = 170.7, 157.8 and 157.7, 153.6 (q, *^2^J_CF_* = 32.4 Hz), 146.1 and 146.0, 131.5 and 131.2, 130.0, 129.9 and 129.8, 129.3 and 129.1, 128.8, 125.6, 121.8 and 121.3, 119.5 (q, *^1^J_CF_* = 274.3 Hz), 118.4 and 118.2, 115.4 and 114.9, 82.3, 73.0, 27.8, 21.7, 18.3. HRMS (ESI): *m*/*z* calculated for C_22_H_24_F_3_NO_6_S + H^+^ [M + H^+^]: 488.1355. Found: 488.1330.

***tert*-Butyl (*R*)-2-(3-(2,2,2-trifluoro-1-((tosyloxy)imino)ethyl)phenoxy)propanoate ((*R*)-50).** The similar treatment of *tert*-butyl (*R*)-2-(3-(2,2,2-trifluoro-1-(hydroxyimino)ethyl)phenoxy)-propanoate (*R*)-**47** (348 mg, 1.04 mmol) as that just described gave (*R*)-**50** (471 mg, 93%). [α]_D_ +34 (*c* 1, CHCl_3_). ^1^H-NMR (270 MHz, CDCl_3_): *δ* = 7.89 (d, *J* = 8.2 Hz, 2H), 7.35 (m, 3H), 7.02 (m, 2H), 6.93 (s, 1H), 4.61 (q, *J* = 6.7 Hz, 1H), 2.46 (s, 3H), 1.59 (d, *J* = 6.6 Hz, 3H), 1.42 (s, 9H). ^13^C-NMR (67.5 MHz, CDCl_3_): *δ* = 170.7, 157.8 and 157.7, 153.7 (q, *^2^J_CF_* = 33.5 Hz), 146.1 and 146.0, 131.5 and 131.2, 130.0, 129.9 and 129.8, 129.3 and 129.1, 127.8, 125.6, 121.8 and 121.3, 119.5 (q, *^1^J_CF_* = 280.5 Hz), 118.4 and 118.2, 115.4 and 114.9, 82.3, 73.1 and 73.0, 27.8, 21.8, 18.3. HRMS (ESI): *m*/*z* calculated for C_22_H_24_F_3_NO_6_S + H^+^ [M + H^+^]: 488.1355. Found: 488.1330.

***tert*-Butyl (*S*)-2-(2-(2,2,2-trifluoro-1-((tosyloxy)imino)ethyl)phenoxy)propanoate ((*S*)-51).** The similar treatment of *tert*-butyl (*S*)-2-(2-(2,2,2-trifluoro-1-(hydroxyimino)ethyl)phenoxy)-propanoate (*S*)-**48** (300 mg, 0.90 mmol) as that just described gave (*S*)-**51** (412 mg, 94%). [α]_D_ +24 (*c* 1, CHCl_3_). ^1^H-NMR (270 MHz, CDCl_3_): *δ* = 7.88 (d, *J* = 8.2 Hz, 2H), 7.39 (m, 3H), 7.12 (d, *J* = 7.6 Hz, 1H), 6.97 (t, *J* = 7.6 Hz, 1H), 6.72 (d, *J* = 7.6 Hz, 1H), 4.56 (q, *J* = 6.8 Hz, 1H), 2.45 (s, 3H), 1.47 (d, *J* = 6.8 Hz, 3H), 1.39 (s, 9H). ^13^C-NMR (67.5 MHz, CDCl_3_): *δ* = 170.3, 156.6, 155.3 (q, *^2^J_CF_* = 34.6 Hz), 145.7, 132.9, 132.4, 131.7, 131.1, 129.8, 129.0, 121.1, 117.8, 116.8 (q, *^1^J_CF_* = 283.2 Hz), 111.8, 82.2, 73.6, 27.7, 21.7, 17.8. HRMS (ESI): *m*/*z* calculated for C_22_H_24_F_3_NO_6_S + H^+^ [M + H^+^]: 488.1355. Found: 488.1339.

***tert*-Butyl (*R*)-2-(2-(2,2,2-trifluoro-1-((tosyloxy)imino)ethyl)phenoxy)propanoate ((*R*)-51).** The similar treatment of *tert*-butyl (*R*)-2-(2-(2,2,2-trifluoro-1-(hydroxyimino)ethyl)phenoxy)-propanoate (*R*)-**48** (254 mg, 0.76 mmol) as that just described gave (*R*)-**51** (373 mg, quant). [α]_D_ −24 (*c* 1, CHCl_3_). ^1^H-NMR (270 MHz, CDCl_3_): *δ* = 7.88 (d, *J* = 8.2 Hz, 2H), 7.39 (m, 3H), 7.12 (d, *J* = 7.6 Hz, 1H), 6.97 (t, *J* = 7.6 Hz, 1H), 6.72 (d, *J* = 7.6 Hz, 1H), 4.55 (q, *J* = 6.8 Hz, 1H), 2.46 (s, 3H), 1.47 (d, *J* = 6.8 Hz, 3H), 1.39 (s, 9H). ^13^C-NMR (67.5 MHz, CDCl_3_): *δ* = 170.3, 156.6, 155.3 (q, *^2^J_CF_* = 35.2 Hz), 145.7, 132.9, 132.4, 131.7, 131.1, 129.8, 129.1, 121.1, 117.8, 116.8 (q, *^1^J_CF_* = 282.7 Hz), 111.8, 82.2, 73.6, 27.8, 21.7, 17.8. HRMS (ESI): *m*/*z* calculated for C_22_H_24_F_3_NO_6_S + H^+^ [M + H^+^]: 488.1355. Found: 488.1362.

***tert*-Butyl (*S*)-2-(4-(3-(trifluoromethyl)diaziridin-3-yl)phenoxy)propanoate ((*S*)-52).** To liquid NH_3_ (10 mL) at −78 °C in a sealed tube, *tert*-butyl (*S*)-2-(2-(2,2,2-trifluoro-1-((tosyloxy)imino)-ethyl)phenoxy)propanoate (*S*)-**49** (336 mg, 0.69 mmol) in dry ether (3 mL) was added. The reaction mixture was stirred at room temperature for 3 h. After evaporation of NH_3_ gas, the reaction mixture was partitioned between ether and water. The organic layer was dried over MgSO_4_, filtrated and concentrated. The residue was purified by column chromatography (ethyl acetate/*n*-hexane, 1:5) to give (*S*)-**52** (200 mg, 88%). [α]_D_ −32 (*c* 1, CHCl_3_). ^1^H-NMR (270 MHz, CDCl_3_): *δ* = 7.51 (d, *J* = 8.6 Hz, 2H), 6.88 (d, *J* = 8.6 Hz, 2H), 4.64 (q, *J* = 6.8 Hz, 1H), 2.76 (d, *J* = 8.6 Hz, 1H), 2.18 (d, *J* = 8.6 Hz, 1H), 1.59 (d, *J* = 6.8 Hz, 3H), 1.44 (s, 9H). ^13^C-NMR (67.5 MHz, CDCl_3_): *δ* = 170.9, 159.0, 129.4, 124.3, 123.6 (q, *^1^J_CF_* = 278.2 Hz), 115.1, 82.1, 72.8, 57.5 (q, *^2^J_CF_* = 35.8 Hz), 27.9, 18.3. HRMS (ESI): *m*/*z* calculated for C_15_H_19_F_3_N_2_O_3_ + H^+^ [M + H^+^]: 333.1426. Found: 333.1414.

***tert*-Butyl (*R*)-2-(4-(3-(trifluoromethyl)diaziridin-3-yl)phenoxy)propanoate ((*R*)-52).** The similar treatment of *tert*-butyl (*R*)-2-(4-(2,2,2-trifluoro-1-((tosyloxy)imino)ethyl)phenoxy)-propanoate (*R*)-**49** (433 mg, 0.89 mmol) as that just described gave (*R*)-**52** (259 mg, 88%). [α]_D_ +32 (*c* 1, CHCl_3_). ^1^H-NMR (270 MHz, CDCl_3_): *δ* = 7.52 (d, *J* = 8.6 Hz, 2H), 6.88 (d, *J* = 8.6 Hz, 2H), 4.64 (q, *J* = 6.8 Hz, 1H), 2.75 (s, 1H), 2.16 (s, 1H), 1.59 (d, *J* = 6.8 Hz, 3H), 1.44 (s, 9H). ^13^C-NMR (67.5 MHz, CDCl_3_): *δ* = 170.9, 159.0, 129.5, 124.3, 123.6 (q, *^1^J_CF_* = 277.7 Hz), 115.1, 82.2, 72.8, 57.6 (q, *^2^J_CF_* = 35.2 Hz), 27.9, 18.3. HRMS (ESI): *m*/*z* calculated for C_15_H_19_F_3_N_2_O_3_ + H^+^ [M + H^+^]: 333.1426. Found: 333.1416.

***tert*-Butyl (*S*)-2-(3-(3-(trifluoromethyl)diaziridin-3-yl)phenoxy)propanoate ((*S*)-53).** The similar treatment of *tert*-butyl (*S*)-2-(3-(2,2,2-trifluoro-1-((tosyloxy)imino)ethyl)phenoxy)-propanoate (*S*)-**50** (431 mg, 0.88 mmol) as that just described gave (*S*)-**53** (269 mg, 92%). [α]_D_ −38 (*c* 1, CHCl_3_). ^1^H-NMR (270 MHz, CDCl_3_): *δ* = 7.32 (t, *J* = 7.6 Hz, 1H), 7.21 (d, *J* = 7.6 Hz, 1H), 7.12 (s, 1H), 6.94 (d, *J* = 7.6 Hz, 1H), 4.64 (q, *J* = 6.8 Hz, 1H), 2.75 (s, 1H), 2.20 (s, 1H), 1.59 (d, *J* = 6.8 Hz, 3H), 1.44 (s, 9H). ^13^C-NMR (67.5 MHz, CDCl_3_): *δ* = 170.9, 157.9, 133.1, 129.9, 123.5 (q, *^1^J_CF_* = 278.2 Hz), 120.9, 116.9, 114.6, 82.2, 72.9, 57.9 (q, *^2^J_CF_* = 35.8 Hz), 27.9, 18.3. HRMS (ESI): *m*/*z* calculated for C_15_H_19_F_3_N_2_O_3_ + H^+^ [M + H^+^]: 333.1426. Found: 333.1414.

***tert*-Butyl (*R*)-2-(3-(3-(trifluoromethyl)diaziridin-3-yl)phenoxy)propanoate ((*R*)-53).** The similar treatment of *tert*-butyl (*R*)-2-(3-(2,2,2-trifluoro-1-((tosyloxy)imino)ethyl)phenoxy)-propanoate (*R*)-**50** (471 mg, 0.97 mmol) as that just described gave (*R*)-**53** (347 mg, quant). [α]_D_ +38 (*c* 1, CHCl_3_). ^1^H-NMR (270 MHz, CDCl_3_): *δ* = 7.32 (t, *J* = 7.6 Hz, 1H), 7.21 (d, *J* = 7.6 Hz, 1H), 7.12 (s, 1H), 6.94 (d, *J* = 7.6 Hz, 1H), 4.64 (q, *J* = 6.8 Hz, 1H), 2.74 (s, 1H), 2.18 (s, 1H), 1.59 (d, *J* = 6.8 Hz, 3H), 1.44 (s, 9H). ^13^C-NMR (67.5 MHz, CDCl_3_): *δ* = 170.9, 157.9, 133.1, 129.9, 123.5 (q, *^1^J_CF_* = 278.2 Hz), 120.9, 116.8, 114.6, 82.1, 72.8, 57.9 (q, *^2^J_CF_* = 35.8 Hz), 27.9, 18.4. HRMS (ESI): *m*/*z* calculated for C_15_H_19_F_3_N_2_O_3_ + H^+^ [M + H^+^]: 333.1426. Found: 333.1421.

***tert*-Butyl (*S*)-2-(2-(3-(trifluoromethyl)diaziridin-3-yl)phenoxy)propanoate ((*S*)-54).** The similar treatment of *tert*-butyl (*S*)-2-(2-(2,2,2-trifluoro-1-((tosyloxy)imino)ethyl)phenoxy)-propanoate (*S*)-**51** (354 mg, 0.73 mmol) as that just described gave (*S*)-**54** (182 mg, 76%). [α]_D_ −17 (*c* 1, CHCl_3_). ^1^H-NMR (270 MHz, CDCl_3_): *δ* = 7.54 (d, *J* = 7.6 Hz, 1H), 7.34 (t, *J* = 7.6 Hz, 1H), 7.01 (t, *J* = 7.6 Hz, 1H), 6.80 (d, *J* = 7.6 Hz, 1H), 4.79 (q, *J* = 6.8 Hz, 1H), 1.63 (d, *J* = 6.8 Hz, 3H), 1.36 (s, 9H). ^13^C-NMR (67.5 MHz, CDCl_3_): *δ* = 170.9, 156.4, 131.5, 130.9, 123.6 (q, *^1^J_CF_* = 278.2 Hz), 121.1, 120.8, 111.9, 82.3, 72.7, 55.8 (q, *^2^J_CF_* = 37.6 Hz), 27.6, 18.2. HRMS (ESI): *m*/*z* calculated for C_15_H_19_F_3_N_2_O_3_ + H^+^ [M + H^+^]: 333.1426. Found: 333.1425.

***tert*-Butyl (*R*)-2-(2-(3-(trifluoromethyl)diaziridin-3-yl)phenoxy)propanoate ((*R*)-54).** The similar treatment of *tert*-butyl (*R*)-2-(2-(2,2,2-trifluoro-1-((tosyloxy)imino)ethyl)phenoxy)-propanoate (*R*)-**51** (317 mg, 0.65 mmol) as that just described gave (*R*)-**54** (173 mg, 80%). [α]_D_ +17 (*c* 1, CHCl_3_). ^1^H-NMR (270 MHz, CDCl_3_): *δ* = 7.54 (d, *J* = 7.6 Hz, 1H), 7.34 (t, *J* = 7.6 Hz, 1H), 7.01 (t, *J* = 7.6 Hz, 1H), 6.80 (d, *J* = 7.6 Hz, 1H), 4.79 (q, *J* = 6.8 Hz, 1H), 1.63 (d, *J* = 6.8 Hz, 3H), 1.36 (s, 9H). ^13^C-NMR (67.5 MHz, CDCl_3_): *δ* = 170.9, 156.4, 131.5, 130.9, 123.6 (q, *^1^J_CF_* = 278.8 Hz), 121.1, 120.8, 111.9, 82.3, 72.7, 55.8 (q, *^2^J_CF_* = 37.4 Hz), 27.6, 18.2. HRMS (ESI): *m*/*z* calculated for C_15_H_19_F_3_N_2_O_3_ + H^+^ [M + H^+^]: 333.1426. Found: 333.1415.

***tert*-Butyl (*S*)-2-(4-(3-(trifluoromethyl)-3*H*-diazirin-3-yl)phenoxy)propanoate ((*S*)-55).***tert*-Butyl (*S*)-2-(4-(3-(trifluoromethyl)diaziridin-3-yl)phenoxy)propanoate (*S*)-**52** (151 mg, 0.45 mmol) was dissolved in CH_2_Cl_2_ (2 mL). MnO_2_ (197 mg, 2.27 mmol) was added to the solution, and the reaction mixture was stirred at room temperature for 2 h, followed by filtration and then concentrated. The residue was purified by column chromatography (ethyl acetate/*n*-hexane, 1:9) to give (*S*)-**55** (136 mg, 91%). [α]_D_ −35 (*c* 1, CHCl_3_). ^1^H-NMR (270 MHz, CDCl_3_): *δ* = 7.12 (d, *J* = 8.6 Hz, 2H), 6.86 (d, *J* = 8.6 Hz, 2H), 4.62 (q, *J* = 6.8 Hz, 1H), 1.58 (d, *J* = 6.8 Hz, 3H), 1.43 (s, 9H). ^13^C-NMR (67.5 MHz, CDCl_3_): *δ* = 170.8, 158.8, 128.1, 122.2 (q, *^1^J_CF_* = 274.9 Hz), 121.6, 115.3, 82.2, 72.9, 28.2 (q, *^2^J_CF_* = 40.8 Hz), 27.9, 18.3. HRMS (ESI): *m*/*z* calculated for C_15_H_17_F_3_N_2_O_3_ + H^+^ [M + H^+^]: 331.1270. Found: 333.1261. Chiral HPLC (*n*-hexane/2-propanol 90:10): *t*_R_ 15.4 min.

***tert*-Butyl (*R*)-2-(4-(3-(trifluoromethyl)-3*H*-diazirin-3-yl)phenoxy)propanoate ((*R*)-55).** The similar treatment of *tert*-butyl (*R*)-2-(4-(3-(trifluoromethyl)diaziridin-3-yl)phenoxy)-propanoate (*R*)-**52** (201 mg, 0.60 mmol) as that just described gave (*R*)-**55** (184 mg, 92%). [α]_D_ +35 (*c* 1, CHCl_3_). ^1^H-NMR (270 MHz, CDCl_3_): *δ* = 7.12 (d, *J* = 8.6 Hz, 2H), 6.86 (d, *J* = 8.6 Hz, 2H), 4.61 (q, *J* = 6.8 Hz, 1H), 1.58 (d, *J* = 6.8 Hz, 3H), 1.43 (s, 9H). ^13^C-NMR (67.5 MHz, CDCl_3_): *δ* = 170.8, 158.8, 128.1, 122.2 (q, *^1^J_CF_* = 274.9 Hz), 121.6, 115.3, 82.2, 72.9, 28.2 (q, *^2^J_CF_* = 40.8 Hz), 27.9, 18.3. HRMS (ESI): *m*/*z* calculated for C_15_H_17_F_3_N_2_O_3_ + H^+^ [M + H^+^]: 331.1270. Found: 333.1260. Chiral HPLC (*n*-hexane/2-propanol 90:10): *t*_R_ 17.1 min.

***tert*-Butyl (*S*)-2-(3-(3-(trifluoromethyl)-3*H*-diazirin-3-yl)phenoxy)propanoate ((*S*)-56).** The similar treatment of *tert*-butyl (*S*)-2-(3-(3-(trifluoromethyl)diaziridin-3-yl)phenoxy)-propanoate (*S*)-**53** (269 mg, 0.81 mmol) as that just described gave (*S*)-**56** (220 mg, 82%). [α]_D_ −38 (*c* 1, CHCl_3_). ^1^H-NMR (270 MHz, CDCl_3_): *δ* = 7.3 (t, *J* = 7.6 Hz, 1H), 6.9 (d, *J* = 7.6 Hz, 1H), 6.8 (d, *J* = 7.6 Hz, 1H), 6.7 (s, 1H), 4.6 (q, *J* = 6.8 Hz, 1H), 1.6 (d, *J* = 6.8 Hz, 3H), 1.4 (s, 9H). ^13^C-NMR (67.5 MHz, CDCl_3_): *δ* = 170.8, 158.0, 130.5, 130.0, 122.1 (q, *^1^J_CF_* = 274.9 Hz), 119.3, 116.3, 113.2, 82.3, 72.9, 28.3 (q, *^2^J_CF_* = 40.8 Hz), 27.9, 18.3. HRMS (ESI): *m/z* calculated for C_15_H_17_F_3_N_2_O_3_ + H^+^ [M + H^+^]: 331.1270. Found: 333.1268. Chiral HPLC (*n*-hexane/2-propanol 90:10): *t*_R_ 9.4 min.

***tert*-Butyl (*R*)-2-(3-(3-(trifluoromethyl)-3*H*-diazirin-3-yl)phenoxy)propanoate ((*R*)-56).** The similar treatment of *tert*-butyl (*R*)-2-(3-(3-(trifluoromethyl)diaziridin-3-yl)phenoxy)-propanoate (*R*)-**53** (347 mg, 1.05 mmol) as that just described gave (*R*)-**56** (283 mg, 82%). [α]_D_ +38 (*c* 1, CHCl_3_). ^1^H-NMR (270 MHz, CDCl_3_): *δ* = 7.3 (t, *J* = 7.6 Hz, 1H), 6.9 (d, *J* = 7.6 Hz, 1H), 6.8 (d, *J* = 7.6 Hz, 1H), 6.7 (s, 1H), 4.6 (q, *J* = 6.8 Hz, 1H), 1.6 (d, *J* = 6.8 Hz, 3H), 1.4 (s, 9H). ^13^C-NMR (67.5 MHz, CDCl_3_): *δ* = 170.8, 158.0, 130.5, 130.0, 122.1 (q, *^1^J_CF_* = 274.9 Hz), 119.3, 116.3, 113.2, 82.3, 72.9, 28.3 (q, *^2^J_CF_* = 40.2 Hz), 27.9, 18.3. HRMS (ESI): *m*/*z* calculated for C_15_H_17_F_3_N_2_O_3_ + H^+^ [M + H^+^]: 331.1270. Found: 333.1263. Chiral HPLC (*n*-hexane/2-propanol 90:10): *t*_R_ 8.8 min.

***tert*-Butyl (*S*)-2-(2-(3-(trifluoromethyl)-3*H*-diazirin-3-yl)phenoxy)propanoate ((*S*)-57).** The similar treatment of *tert*-butyl (*S*)-2-(2-(3-(trifluoromethyl)diaziridin-3-yl)phenoxy)-propanoate (*S*)-**54** (105 mg, 0.32 mmol) as that just described gave (*S*)-**57** (93.4 mg, 89%). [α]_D_ −10 (*c* 1, CHCl_3_). ^1^H-NMR (270 MHz, CDCl_3_): *δ* = 7.37 (d, *J* = 7.6 Hz, 1H), 7.24 (t, *J* = 7.6 Hz, 1H), 6.87 (t, *J* = 7.6 Hz, 1H), 6.66 (d, *J* = 7.6 Hz, 1H), 4.60 (q, *J* = 6.8 Hz, 1H), 1.61 (d, *J* = 6.8 Hz, 3H), 1.30 (s, 9H). ^13^C-NMR (67.5 MHz, CDCl_3_): *δ* = 170.7, 158.0, 131.7, 131.1, 122.1 (q, *^1^J_CF_* = 274.9 Hz), 121.3, 117.1, 112.2, 82.1, 73.3, 27.8, 26.4 (q, *^2^J_CF_* = 43.0 Hz), 18.2. HRMS (ESI): *m*/*z* calculated for C_15_H_17_F_3_N_2_O_3_ + H^+^ [M + H^+^]: 331.1270. Found: 333.1285. Chiral HPLC (*n*-hexane/2-propanol 90:10): *t*_R_ 9.9 min.

***tert*-Butyl (*R*)-2-(2-(3-(trifluoromethyl)-3*H*-diazirin-3-yl)phenoxy)propanoate ((*R*)-57).** The similar treatment of *tert*-butyl (*R*)-2-(2-(3-(trifluoromethyl)diaziridin-3-yl)phenoxy)-propanoate (*R*)-**54** (85.8 mg, 0.26 mmol) as that just described gave (*R*)-**57** (81.7 mg, 96%). [α]_D_ +10 (*c* 1, CHCl_3_). ^1^H-NMR (270 MHz, CDCl_3_): *δ* = 7.45 (d, *J* = 7.6 Hz, 1H), 7.33 (t, *J* = 7.6 Hz, 1H), 6.96 (t, *J* = 7.6 Hz, 1H), 6.74 (d, *J* = 7.6 Hz, 1H), 4.68 (q, *J* = 6.8 Hz, 1H), 1.69 (d, *J* = 6.8 Hz, 3H), 1.39 (s, 9H). ^13^C-NMR (67.5 MHz, CDCl_3_): *δ* = 170.7, 158.0, 131.7, 131.1, 122.1 (q, *^1^J_CF_* = 274.9 Hz), 121.3, 117.1, 112.2, 82.1, 73.2, 27.8, 26.4 (q, *^2^J_CF_* = 43.0 Hz), 18.2. HRMS (ESI): *m*/*z* calculated for C_15_H_17_F_3_N_2_O_3_ + H^+^ [M + H^+^]: 331.1270. Found: 333.1276. Chiral HPLC (*n*-hexane/2-propanol 90:10): *t*_R_ 10.3 min.

**(*S*)-2-(4-(3-(Trifluoromethyl)-3*H*-diazirin-3-yl)phenoxy)propanoic acid ((*S*)-58).***tert*-Butyl (*S*)-2-(4-(3-(trifluoromethyl)-3*H*-diazirin-3-yl)phenoxy)propanoate (*S*)-**55** (165 mg, 0.50 mmol) was dissolved in CH_2_Cl_2_ (1 mL) and then trifluoroacetic acid (2 mL) was added to the solution. After the reaction mixture was stirred at room temperature for 2 h, the reaction mixture was poured into the cold water and extracted by CH_2_Cl_2_. The organic layer was dried over MgSO_4_, filtrated and concentrated to give (*S*)-**58** (135 mg, 98%). [α]_D_ −20 (*c* 1, CHCl_3_). ^1^H-NMR (270 MHz, CDCl_3_): *δ* = 11.33 (s, 1H), 7.14 (d, *J* = 8.6 Hz, 2H), 6.89 (d, *J* = 8.6 Hz, 2H), 4.79 (q, *J* = 6.8 Hz, 1H), 1.66 (d, *J* = 6.8 Hz, 3H). ^13^C-NMR (67.5 MHz, CDCl_3_): *δ* = 177.8, 158.2, 128.3, 122.3, 122.2 (q, *^1^J_CF_* = 274.3 Hz), 115.4, 71.9, 28.1 (q, *^2^J_CF_* = 40.8 Hz), 18.3. HRMS (ESI): *m*/*z* calculated for C_11_H_9_F_3_N_2_O_3_ + H^+^ [M + H^+^]: 275.0644. Found: 275.0648.

**(*R*)-2-(4-(3-(Trifluoromethyl)-3*H*-diazirin-3-yl)phenoxy)propanoic acid ((*R*)-58).** The similar treatment of *tert*-butyl (*R*)-2-(4-(3-(trifluoromethyl)-3*H*-diazirin-3-yl)phenoxy)-propanoate (*R*)-**55** (201 mg, 0.61 mmol) as that just described gave (*R*)-**58** (184 mg, 92%). [α]_D_ +20 (*c* 1, CHCl_3_). ^1^H-NMR (270 MHz, CDCl_3_): *δ* = 10.28 (s, 1H), 7.15 (d, *J* = 8.6 Hz, 2H), 6.89 (d, *J* = 8.6 Hz, 2H), 4.79 (q, *J* = 6.8 Hz, 1H), 1.67 (d, *J* = 6.8 Hz, 3H). ^13^C-NMR (67.5 MHz, CDCl_3_): *δ* = 177.4, 158.2, 128.3, 122.3, 122.2 (q, *^1^J_CF_* = 274.3 Hz), 115.4, 72.0, 28.1 (q, *^2^J_CF_* = 40.8 Hz), 18.3. HRMS (ESI): *m*/*z* calculated for C_11_H_9_F_3_N_2_O_3_ + H^+^ [M + H^+^]: 275.0644. Found: 275.0614.

**(*S*)-2-(3-(3-(Trifluoromethyl)-3*H*-diazirin-3-yl)phenoxy)propanoic acid ((*S*)-59).** The similar treatment of *tert*-butyl (*S*)-2-(3-(3-(trifluoromethyl)-3*H*-diazirin-3-yl)phenoxy)-propanoate (*S*)-**56** (220 mg, 0.67 mmol) as that just described gave (*S*)-**59** (209 mg, quant). [α]_D_ −13 (*c* 1, CHCl_3_). ^1^H-NMR (270 MHz, CDCl_3_): *δ* = 11.47 (s, 1H), 7.29 (t, *J* = 7.8 Hz, 1H), 6.89 (d, *J* = 7.8 Hz, 1H), 6.79 (d, *J* = 7.8 Hz, 1H), 6.74 (s, 1H), 4.78 (q, *J* = 6.8 Hz, 1H), 1.66 (d, *J* = 6.8 Hz, 3H). ^13^C-NMR (67.5 MHz, CDCl_3_): *δ* = 178.0, 157.5, 130.9, 130.2, 122.0 (q, *^1^J_CF_* = 274.7 Hz), 119.8, 115.7, 114.0, 72.1, 28.3 (q, *^2^J_CF_* = 39.9 Hz), 18.2. HRMS (ESI): *m*/*z* calculated for C_11_H_9_F_3_N_2_O_3_ + H^+^ [M + H^+^]: 275.0644. Found: 275.0661.

**(*R*)-2-(3-(3-(Trifluoromethyl)-3*H*-diazirin-3-yl)phenoxy)propanoic acid ((*R*)-59).** The similar treatment of *tert*-butyl (*R*)-2-(3-(3-(trifluoromethyl)-3*H*-diazirin-3-yl)phenoxy)-propanoate (*R*)-**56** (283 mg, 0.86 mmol) as that just described gave (*R*)-**59** (208 mg, 89%). [α]_D_ +13 (*c* 1, CHCl_3_). ^1^H-NMR (270 MHz, CDCl_3_): *δ* = 11.32 (s, 1H), 7.30 (t, *J* = 8 Hz, 1H), 6.90 (d, *J* = 7.8 Hz, 1H), 6.80 (d, *J* = 7.8 Hz, 1H), 6.74 (s, 1H), 4.78 (q, *J* = 6.8 Hz, 1H), 1.66 (d, *J* = 6.8 Hz, 3H). ^13^C-NMR (67.5 MHz, CDCl_3_): *δ* = 177.9, 157.5, 130.9, 130.2, 122.0 (q, *^1^J_CF_* = 274.5 Hz), 119.8, 115.7, 114.0, 72.1, 28.3 (q, *^2^J_CF_* = 39.7 Hz), 18.2. HRMS (ESI): *m*/*z* calculated for C_11_H_9_F_3_N_2_O_3_ + H^+^ [M + H^+^]: 275.0644. Found: 275.0627.

**(*S*)-2-(2-(3-(Trifluoromethyl)-3*H*-diazirin-3-yl)phenoxy)propanoic acid ((*S*)-60).** The similar treatment of *tert*-butyl (*S*)-2-(2-(3-(trifluoromethyl)-3*H*-diazirin-3-yl)phenoxy)-propanoate (*S*)-**57** (95.4 mg, 0.29 mmol) as that just described gave (*S*)-**60** (89.0 mg, quant). [α]_D_ −10 (*c* 1, CHCl_3_). ^1^H-NMR (270 MHz, CDCl_3_): *δ* = 7.49 (d, *J* = 7.6 Hz, 1H), 7.37 (t, *J* = 7.6 Hz, 1H), 7.02 (t, *J* = 7.6 Hz, 1H), 6.78 (d, *J* = 7.6 Hz, 1H), 4.86 (q, *J* = 6.8 Hz, 1H), 1.78 (d, *J* = 6.8 Hz, 3H). ^13^C-NMR (67.5 MHz, CDCl_3_): *δ* = 177.1, 157.5, 132.0, 131.4 122.0 (q, *^1^J_CF_* = 275.4 Hz), 122.0, 117.4, 112.4, 72.3, 26.3 (q, *^2^J_CF_* = 42.5 Hz), 18.2. HRMS (ESI): *m*/*z* calculated for C_11_H_9_F_3_N_2_O_3_ + H^+^ [M + H^+^]: 275.0644. Found: 275.0663.

**(*R*)-2-(2-(3-(Trifluoromethyl)-3*H*-diazirin-3-yl)phenoxy)propanoic acid ((*R*)-60).** The similar treatment of *tert*-butyl (*R*)-2-(2-(3-(trifluoromethyl)-3*H*-diazirin-3-yl)phenoxy)-propanoate (*R*)-**57** (166 mg, 0.50 mmol) as that just described gave (*R*)-**60** (151 mg, quant). [α]_D_ +10 (*c* 1, CHCl_3_). ^1^H-NMR (270 MHz, CDCl_3_): *δ* = 7.50 (d, *J* = 7.6 Hz, 1H), 7.38 (t, *J* = 7.6 Hz, 1H), 7.03 (t, *J* = 7.6 Hz, 1H), 6.80 (d, *J* = 7.6 Hz, 1H), 4.88 (q, *J* = 6.8 Hz, 1H), 1.77 (d, *J* = 6.8 Hz, 3H). ^13^C-NMR (67.5 MHz, CDCl_3_): *δ* = 176.6, 157.4, 132.0, 131.3, 122.0, 122.0 (q, *^1^J_CF_* = 274.9 Hz), 117.3, 112.3, 72.2, 26.3 (q, *^2^J_CF_* = 42.5 Hz), 18.1. HRMS (ESI): *m*/*z* calculated for C_11_H_9_F_3_N_2_O_3_ + H^+^ [M + H^+^]: 275.0644. Found: 275.0654.

### 4.6. Cell-Based Sweet Taste Assay for Photoreactive Lactisole Derivatives

**Construction of cell lines stably expressing the sweet taste receptor.** The previously reported method to construct a cell line stably expressing the human sweet taste receptor [37,38] was also used in this study. The genes encoding T1R2, T1R3, and Gα16gust44 were incorporated into a modified version of the pcDNA5/FRT vector (Thermo Fisher Scientific, Waltham, MA, USA.). Following the Flp-In System protocol (Thermo Fisher Scientific, Waltham, MA, USA.), this construct was transfected into Flp-In 293 cells.

**Measurement of cellular responses.** Measurements of cellular responses were performed as described previously [5,37,38]. At first, all synthesized compounds were prepared in 1 M solution using dimethyl sulfoxide, and diluted by assay buffer. Cellular responses administrated mixtures containing both 1 mM aspartame and various concentrations of inhibitors were measured. Flp-In 293 cells stably expressing the sweet taste receptor were seeded in 96-well plates and incubated for an additional 23 h. The cells were washed with assay buffer and loaded using the FRIPR Calcium 4 Assay Kit (Molecular Devices, San Jose, CA, USA.). Measurement was performed on FlexStation 3 (Molecular Devices, San Jose, CA, USA.) after the samples were incubated at 37 °C for 1 h. The temperature of the FlexStation 3 was also maintained at 37 °C.

**Data analysis.** All data were normalized by the cellular response against 1 mM aspartame and were fitted to Hill’s equation, which was drawn using Clampfit 9.2 (Molecular Devices, Palo Alto, CA, USA), and IC_50_ values were calculated from a dose–response curve.

## Figures and Tables

**Table 1:** Inhibitory activities of photophore containing lactisole derivatives. IC_50_: 50% inhibitory concentration.

Position of Photophore	Benzophenone	IC_50_ (µM)	Azide	IC_50_ (µM)	Diazirine	IC_50_ (µM)
4-	(*S*)-**9**	970 ± 11	(*S*)-**20**	100 ± 0.77	(*S*)-**58**	12 ± 0.067
	(*R*)-**9**	2200 ± 32	(*R*)-**20**	1800 ± 24	(*R*)-**58**	250 ± 1.8
3-	(*S*)-**10**	1100 ± 10	(*S*)-**21**	100 ± 0.83	(*S*)-**59**	19 ± 0.14
	(*R*)-**10**	1100 ± 11	(*R*)-**21**	2400 ± 23	(*R*)-**59**	250 ± 1.7
2-	(*S*)-**11**	1200 ± 20	(*S*)-**28**	280 ± 5.2	(*S*)-**60**	37 ± 0.28
	(*R*)-**11**	1200 ±18	(*R*)-**28**	1200 ± 18	(*R*)-**60**	300 ± 1.8
Control	*rac*-lactisole **61**	82 ± 0.44	(*S*)-**61**	20 ± 4.3	(*R*)-**61**	nd
	*rac*-2,4-DP **62**	7.9 ± 0.067	(*S*)-**62**	2.6 ± 0.51	(*R*)-**62**	45 ± 6.1

nd: not determined.

## Supplementary Materials

The following are available online, ^1^H- and ^13^C- data for synthetic compounds, Cellular response of each photophore containing lactisole derivatives.
